# Cerebral Oximetry–Guided Treatment and Cerebral Oxygenation in Extremely Preterm Infants: A Randomized Clinical Trial

**DOI:** 10.1001/jamanetworkopen.2025.57620

**Published:** 2026-02-05

**Authors:** Pranav R. Jani, Traci-Anne Goyen, Kiran Kumar Balegar, Rajesh Maheshwari, Maria Saito-Benz, Tim Schindler, James Moore, Manelle Merhi, Melinda Cruz, Yang Song, Hayley McDonagh, Melissa Luig, Mark Tracy, Daphne D’Cruz, Aldo Perdomo, Stephanie Morakeas, Vishnu Dasireddy, Mihaela Culcer, Vijay Shingde, Karen Bennington, Joanna Michalowski, Andreja Fucek, Jennifer Querim, Sean Stevens, James Santanelli, James Elhindi, Brian Gloss, Robert Halliday, Dharmesh Shah, Himanshu Popat

**Affiliations:** 1 Department of Neonatology, Westmead Hospital, Westmead, New South Wales, Australia; 2 Faculty of Medicine and Health, The University of Sydney, Sydney, New South Wales, Australia; 3 Department of Neonatology, Nepean Hospital, Nepean Blue Mountains Local Health District, Kingswood, Australia; 4 Sydney Medical School Nepean, The University of Sydney, Sydney, New South Wales, Australia; 5 Neonatal Intensive Care Unit, Wellington Regional Hospital, Wellington, New Zealand; 6 Department of Paediatrics and Child Health, University of Otago, Wellington, New Zealand; 7 Department of Newborn Care, The Royal Hospital for Women, Sydney, New South Wales, Australia; 8 School of Clinical Medicine, University of New South Wales, Sydney, New South Wales, Australia; 9 Connecticut Children’s, Division of Neonatal-Perinatal Medicine, Connecticut Children’s Medical Center, Hartford; 10 UConn School of Medicine Farmington, Farmington, Connecticut; 11 Kennards Hire Group, Sydney, Australia; 12 National Health and Medical Research Council Clinical Trial Centre, University of Sydney, Camperdown, New South Wales, Australia; 13 NICU Lived Network, Sydney, Australia; 14 Lived Experience Advisory Network, Perinatal Society of Australia and New Zealand, Mornington, Australia; 15 Research and Education Network, Westmead Hospital, Westmead, New South Wales, Australia; 16 Faculty of Engineering and Information Technologies, Biomedical Engineering Institute, The University of Sydney, Sydney, New South Wales, Australia; 17 Westmead Research Hub, Westmead Institute for Medical Research, Sydney, New South Wales, Australia; 18 Grace Centre for Newborn Intensive Care, The Children’s Hospital for Westmead, Westmead, New South Wales, Australia

## Abstract

**Importance:**

Preterm infants are at high risk of developing brain injury, and near-infrared spectroscopy (NIRS) offers the ability to measure cerebral oxygenation. The impact of using a standardized treatment guideline combined with a single NIRS device manufacturer (Nonin Medical Inc) and neonatal sensor on cerebral oxygenation has not been previously examined.

**Objective:**

To investigate whether cerebral oximetry with a dedicated treatment guideline improves cerebral oxygenation stability.

**Design, Setting, and Participants:**

This was a single-blinded, 2-arm randomized clinical trial conducted from October 2021 to July 2024 at 5 tertiary neonatal intensive care units across Australia, New Zealand, and the US. Infants born at less than 29 weeks’ gestation and aged younger than 6 hours underwent 1:1 random allocation stratified by gestational age (<26 weeks and ≥26 weeks) and study site.

**Intervention:**

The intervention group received cerebral oximetry and dedicated guideline-based treatment when cerebral oxygenation was outside the range of 65% to 90%. The control group had blinded cerebral oximetry and treatment guided by standard clinical monitoring.

**Main Outcomes and Measures:**

The burden of cerebral hypoxia and hyperoxia during the first 5 days after birth expressed as percentage hours was the primary outcome. Key secondary outcomes were mortality, morbidities before discharge, and NIRS-related skin injury.

**Results:**

Of 149 screened infants (53 randomized to the intervention and 51 randomized to standard care), 100 infants were included in the final analysis (median [IQR] gestational age, 27 [25-28] weeks; 48 male [48.0%]). The median (IQR) birth weight was 883 (709-1079) g. The intervention group (50 infants) had a significantly lower median (IQR) burden of hypoxia and hyperoxia of 5.7% hours (2.8% hours to 15.0% hours) compared with 39.6% hours (6.5% hours to 82.3% hours) in the standard care group (50 infants), with an adjusted reduction of 42.8% hours (95% CI, 35.6% hours to 53.3% hours; *P* < .001). Mortality, morbidities before discharge, and safety outcomes were comparable between groups.

**Conclusions and Relevance:**

In this study, treatment guided by cerebral oximetry with a single device manufacturer and a neonatal sensor significantly improved the stability of cerebral oxygenation in extremely preterm infants. Larger multicenter trials are warranted to determine if this finding leads to improved survival without brain injury.

**Trial Registration:**

Australian New Zealand Clinical Trials Registry registration number ACTRN12621000778886

## Key Points

**Question:**

Does treatment guided by cerebral oximetry using a near-infrared spectroscopy device from a single manufacturer and a neonatal sensor improve cerebral oxygenation stability in extremely preterm infants?

**Findings:**

In this randomized clinical trial of 100 infants born at less than 29 weeks’ gestation, the burden of cerebral hypoxia and hyperoxia was significantly lower in the intervention group compared with the standard care group.

**Meaning:**

In this study, treatment guided by cerebral oximetry improved the stability of cerebral oxygenation in extremely preterm infants.

## Teaser

This randomized clinical trial investigates the effect of cerebral oximetry using a near-infrared spectroscopy device from a single manufacturer and a neonatal sensor on cerebral oxygenation stability in extremely preterm infants.

## Introduction

Infants born extremely preterm (<28 weeks’ gestation) have an increased risk of death and neurodevelopmental deficits, with consequently substantial societal economic impacts.^1,2,3,4^ They often experience cardiorespiratory instability during the early postnatal period, presenting as episodes of systemic hypoxia, hyperoxia, or both, conditions that are associated with an increased risk of brain injury.^5,6,7^ A meta-analysis^8^ from 5 clinical trials comparing lower (85%-89%) vs higher (91%-95%) peripheral oxygen saturation target ranges in extremely preterm infants reported decreased risk of death and severe necrotizing enterocolitis and increased risk of treated retinopathy of prematurity (ROP) in infants assigned to the higher target ranges but similar rates of death or major disability at corrected ages 18 to 24 months. Targeting the stability of cerebral oxygenation by using near infrared spectroscopy (NIRS; cerebral oximetry) compared with systemic assessment of oxygenation using pulse oximetry offers an alternative physiological approach for reducing brain injury.^9^

The SafeBoosC-II study demonstrated that cerebral NIRS-guided treatment could stabilize cerebral oxygenation in extremely preterm infants.^10^ However, the use of multiple NIRS platforms, modified alarm thresholds, and adult sensors measuring lower absolute oxygenation values compared with neonatal sensors introduced variability in cerebral oxygenation. This trial, Near Infra-Red Spectroscopy Targeted Use to Reduce Adverse Outcomes in Extremely Preterm Infants (NIRTURE), was built on this evidence. It tested the hypothesis that the burden of cerebral hypoxia and hyperoxia could be reduced by combining cerebral oximetry with a dedicated treatment guideline using a NIRS device from 1 manufacturer (SenSmart Model X-100 Universal Oximetry System [Nonin Medical Inc]) and a neonatal sensor compared with blinded cerebral oximetry and standard care during the early postnatal period. Unlike SafeBoosC-II study, this trial extended monitoring from 3 to 5 days postnatally, used real-time and unmodified alarms for detecting cerebral hypoxia and hyperoxia, used a NIRS device from a single manufacturer to reduce interdevice variability, and adopted a pragmatic cerebral oxygenation reference range (65%-90%). The lower threshold (65%) was adapted from the SafeBoosC-II study to reflect higher readings from neonatal sensors, and the upper threshold (90%) was informed by unpublished data from Westmead Hospital (lead site).^10,11^ This trial aims to inform future randomized clinical trials (RCTs) evaluating the impact of this intervention on childhood survival free of neurological deficits.

## Methods

Ethical approvals for this RCT were obtained before trial commencement (Australia: Sydney Children’s Hospitals Network Human Research Ethics Committee; New Zealand: Health and Disability Ethics Committee; US: Institutional Review Board of the Connecticut Children’s Medical Center). The trial was conducted in accordance with the International Council for Harmonisation guidelines for Good Clinical Practice. Results are reported according to the Consolidated Standards of Reporting Trials (CONSORT) reporting guideline. Deferred consent was used at 3 Australian sites, with written consent obtained within 5 days of birth; 2 international sites used prospective written consent, obtained antenatally or within 6 hours postnatally. The trial protocol, including details of the methodology, was previously published (see Supplement 1).^12^

### Consumer and Community Involvement

Parents with a lived experience of preterm birth were actively engaged in codesigning this trial. Their involvement included reviewing and advising on study methodology; contributing to developing the consent process, particularly advocating for deferred consent; and advising the trial management committee on participant information and consent forms. Individual parent and parent support organizations were consulted for the decision to allocate siblings of multiple births to the same treatment group.

### Trial Design and Changes to Trial Protocol

This is a multisite, single-blinded, 2-arm RCT with 1:1 allocation, stratified by gestational age (<26 weeks and ≥26 weeks) and study site. A modification to the trial protocol was implemented to address instances in which participants were randomized but parental consent could not be obtained in the first 5 days after birth. This was due to circumstances such as COVID-19–related isolation or maternal sickness requiring intensive care unit admission. In these cases, consent was later obtained from the family before including participant data in the analysis. No changes were made to predefined outcomes or analyses after trial commencement.

### Trial Setting

This trial was conducted across 5 tertiary units across Australia, New Zealand, and the US. Participating sites included Westmead Hospital, Nepean Hospital, and The Royal Hospital for Women in Australia; Wellington Hospital in New Zealand; and the Connecticut Children’s Hospital in the US. Investigators at each site had prior experience with cerebral oximetry, but most clinical staff at these sites did not. Therefore, clinical staff underwent structured training and completed internet-based certification before site activation and participant recruitment.

### Eligibility Criteria

We included inborn and outborn infants (singleton or twin births) born at less than 29 weeks’ gestation who were aged younger than 6 hours. Exclusion criteria were antenatal or postnatal diagnosis of a congenital anomaly requiring major surgery, genetic disorders associated with neurological impairment, and multiple births beyond twins.

### Randomization

Randomization was performed by research or clinical staff using REDCap (University of Sydney license), with variable block randomization stratified by gestational age and site. An independent biostatistician generated permuted block codes to ensure 1:1 group balance. Twins were assigned to the same group to account for clustering. Withdrawn infants were replaced in the study with new randomization. Data analysts (J.E., B.G., and R.H.) were blinded to group allocation.

### Intervention and Comparator

After enrolment, infants were randomly allocated to the standard care or intervention group. Cerebral oximetry in both groups was performed using a single neonatal NIRS sensor placed on the frontotemporal region on 1 side of the infant’s head at any given time. Cerebral NIRS data were continuously recorded using the SenSmart Model X-100 Universal Oximetry System for 5 days from birth (120 hours). In the intervention group, real-time cerebral oxygenation readings were visible to clinical staff, who managed infants according to a predefined treatment guideline when cerebral oxygenation values were outside the target range of 65% to 90%. In the standard care group, the NIRS monitor screen was covered with an opaque cover to prevent clinical staff from acting on these values. Infants in the standard care group received care according to each participating site’s clinical practice. For example, care could include increasing oxygen delivery in response to low peripheral oxygen saturation. There were no restrictions on any other aspects of concomitant care.

### Intervention Thresholds

A pragmatic, consensus-based approach was used to define the reference range for cerebral oxygenation. The lower threshold of 65% was chosen based on the threshold used in the SafeBoosC-II study and adjusted to account for higher absolute oxygenation values observed with neonatal sensors. The upper threshold of 90% instead of the 85% used in the SafeBoosC-II study was chosen from local, unpublished data from Westmead Hospital. In this cohort, a cerebral oxygenation value of 87% corresponded to the 75th percentile. The sensor site was inspected every 4 hours to ensure correct sensor placement and skin integrity.

A clinical treatment guideline for cerebral hypoxia was activated when cerebral oxygenation was less than 65%, and a clinical treatment guideline for cerebral hyperoxia was activated when cerebral oxygenation was greater than 90% (eFigures 1-4 in Supplement 2). NIRS-guided treatment, including sensor repositioning, was initiated within 1 minute of an alarm, with treatment response reassessed within 30 minutes. Further details on the treatment guideline are available in Jani et al.^12^

### Outcomes

The primary outcome was the burden of cerebral hypoxia and hyperoxia during the first 5 days after birth, expressed as percentage hours. An hour event with a mean cerebral oxygenation of 55% would equate to 10% hours of hypoxia (10% below the lower threshold of 65% multiplied by 1 hour). Only deviations lasting longer than 1 minute were included in the calculation of the primary outcome; this was done to minimize false positive alarms from poor sensor contact while capturing clinically relevant changes in cerebral oxygenation. The primary outcome for events exceeding 10 minutes is reported for comparison with other studies (eTable 1 in Supplement 2).^10^ Secondary outcomes before hospital discharge were mortality, brain injury on imaging (any intraventricular hemorrhage,^13^ cerebellar hemorrhage, or periventricular leukomalacia on ultrasonography, magnetic resonance imaging, or both), chronic lung disease (need for respiratory support or supplemental oxygen at 36 weeks’ postmenstrual age), necrotizing enterocolitis using Bell classification,^14^ and ROP.

### Data Collection and Harms

Deidentified data collection for the previously listed outcomes was done in the REDCap electronic data capture tool (University of Sydney license). Skin injury (pressure or thermal) associated with the NIRS sensor within the first 5 days after birth was monitored as a safety outcome.

### Sample Size

Based on results of the SafeBoosC-II study,^10^ we assumed that a 50% decrease in the primary outcome would represent a clinically meaningful effect. This corresponded to a decrease of 0.3 in the mean of the log-transformed burden of cerebral hypoxia, hyperoxia, or both. Assuming an SD of 0.5 in the log-transformed burden,^15^ a sample size of 45 infants per group could achieve 80% power with a 2-sided α of .05. Accounting for the clustering effect from twin births allocated to the same treatment and based on a regional incidence of twins of approximately 30%, the mean cluster size was estimated at 1.3, with an assumed intracluster correlation of 0.1. This gave a design effect of 1.03, taking the sample size to 47 infants per group or 94 infants in total. To accommodate loss to follow-up or missing data, the final sample size was set at 100 infants.

For consented infants in whom NIRS monitoring was discontinued earlier than the planned 5 days after the birth monitoring period, data collected until discontinuation were included in the primary outcome analysis. Study oversight was provided by an independent Data Safety and Monitoring Committee, which monitored all aspects of the trial conduct every 6 months until recruitment was complete. An interim analysis was not conducted during the trial.

### Statistical Analysis

All statistical analyses were conducted per protocol using RStudio version 4 (RStudio) at a significance level of .05 with a 2-sided alternative hypothesis. No corrections for multiple hypotheses were performed. Participant demographic and clinical characteristics and study outcomes are presented using standard descriptive statistics; for categorical variables, we used frequencies and percentages, and for continuous variables, we used mean with SD and range or median with quartiles and range. All efficacy analyses were performed according to randomized treatment. We used a log-normal transformation to ensure normality for the primary outcome and a linear mixed effects model with clustering by hospital for comparison between groups. Subgroup analysis for gestational age stratum was performed. Other study outcomes were compared using linear mixed effects models with normal, log-normal, or binary outcome distribution. Adjusted models were used to explore predictors of outcomes. Variables that differed significantly between the 2 groups and were plausibly contributory to the outcome were considered for adjustment. A fixed adjustment for the gestational age category was included as an a priori specified variable. Models that additionally included clustering for twins were examined but did not better fit the data as per a likelihood ratio test. A statistical analysis plan is provided in Supplement 1.

## Results

**Figure 1.**
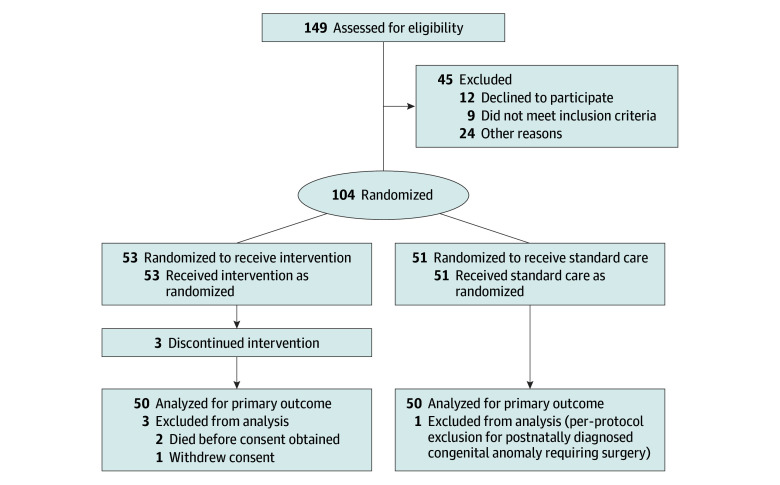
Study Flowchart Flow of study infants is presented as per the updated Consolidated Standards of Reporting Trials (CONSORT) reporting guideline.^16^ Other reasons were missed eligible participants and no research staff after hours for consenting. Recruitment by sites was as follows: 3 participants at site A, 32 participants at site B, 20 participants each at sites C and D, and 25 participants at site E.

Between October 2021 and July 2024, a total of 149 infants were screened for eligibility. Of these, 104 infants were randomized to the intervention group (53 infants) or the standard care group (51 infants) (Figure 1).^16^ After randomization, 4 infants were excluded: 2 infants died within the first 5 days before consent could be obtained (unrelated to the intervention), while 1 infant was diagnosed postnatally with a congenital anomaly requiring surgery and 1 parent withdrew consent. A total of 100 infants (50 individuals in each group) received the allocated intervention and were included in the primary outcome analysis (median [IQR] gestational age, 27 [25-28] weeks; 48 males [48.0%]). One infant in each group died within the first 5 days after birth and was included in final analyses. These deaths were not related to the intervention. Except for these 2 infants, all remaining infants received protocol-defined cerebral oximetry for the first 5 days after birth without loss to follow-up.

Baseline characteristics between the 2 groups were similar; however, the intervention group had a higher proportion of infants who were exposed to chorioamnionitis and who were in larger birth weight percentiles (Table 1). We adjusted for these variables in the primary outcome analysis. The median (IQR) birth weight was 883 (709-1079) g. No significant differences were observed between groups regarding rates of cardiorespiratory treatment, complications, sepsis, or mortality within the first 5 days after birth (eTable 2 in Supplement 2).

**Table 1.** Participant Characteristics

Characteristic	Participants, No. (%) (N = 100)	
	Intervention group (n = 50)^a^	Standard care group (n = 50)^b^
Gestational age at birth, wk		
Median (IQR)	26.9 (25.7-27.7)	27.0 (25.9-28.4)
23^+0^ to 25^+6^	15 (30.0)	14 (28.0)
26^+0^ to 28^+6^	35 (70.0)	36 (72.0)
Sex		
Male	23 (46.0)	25 (50.0)
Female	27 (54.0)	25 (50.0)
Twin births	11 (22.0)	12 (24.0)
Received any antenatal steroids	47 (94.0)	50 (100)
Received magnesium sulfate	45 (90.0)	44 (88.0)
Prolonged rupture of membranes >18 h	16 (32.0)	13 (26.0)
Pathologically confirmed chorioamnionitis^c^	23 (46.0)	11 (22.0)
Cesarean delivery	28 (56.0)	33 (66.0)
Inborn birth	48 (96.0)	49 (98.0)
Apgar score <7 at 5 min	12 (24.0)	11 (22.0)
Birthweight, median (IQR), g	962 (725-1050)	855 (706-1113)
Birthweight (percentiles), median (IQR)^c^	47 (33-77)	34 (3-60)
Intubated at delivery	22 (44.0)	21 (42.0)
Gestational age at discharge, median (IQR), wk^d^	40.4 (38.3-42.9)	40.3 (38.7-42.9)
Weight at discharge, median (IQR), g^d^	2962 (2533-3331)	2958 (2630-3388)

^a^ Cerebral oximetry + clinical treatment guideline.

^b^ Blinded cerebral oximetry + treatment as usual.

^c^ Characteristics with an imbalance.

^d^ Excludes infants who died.

### Primary Outcome

**Figure 2.**
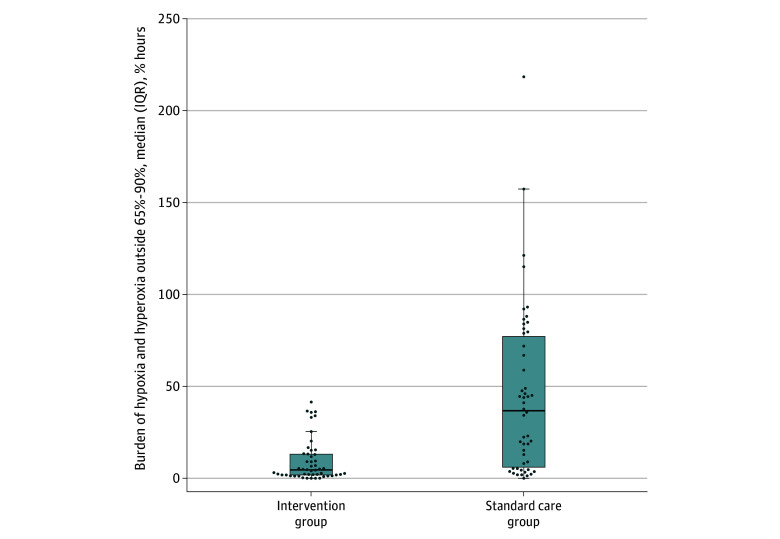
Primary Outcome by Treatment The burden of cerebral hypoxia and hyperoxia outside 65% to 90% is expressed as % hours based on the allocated group. Bold horizontal lines within boxes indicate medians; boxes, IQRs; lower whiskers, hinge to smallest value at most 1.5 × IQR of hinge; dots, individual values; upper whiskers, largest value no further than 1.5 × IQR from the hinge (edge of central box).

Among 50 infants randomized to the intervention group, the combined median (IQR) burden of cerebral hypoxia and hyperoxia was 5.7% hours (2.8% hours to 15.0% hours) compared with 39.6% hours (6.5% hours to 82.3% hours) in 50 infants randomized to the standard care group. The adjusted relative change in the primary outcome for the intervention group was 42.8% (95% CI, 35.6% to 53.3%; *P* < .001) (Figure 2). The median (IQR) burden of cerebral hyperoxia decreased from 23.7% hours (5.8% hours to 60.7% hours) in the standard care group to 3.5% hours (2.0% hours to 14.4% hours) in the intervention group, corresponding to an adjusted relative change of 40.3% hours (95% CI, 30.5% hours to 59.5% hours; *P* = .01). Similarly, the median (IQR) burden of cerebral hypoxia was decreased from 2.5% hours (1.1% hours to 8.0% hours) in the standard care group to 0.7% hours (<0.1% hours to 1.9% hours) in the intervention group, with an adjusted relative change of 335.9% hours (95% CI, 325.7% hours to 1019.0% hours; *P* < .001). The primary outcome was analyzed by gestational age group, where the relative change in burden of cerebral hypoxia and hyperoxia was 57.0% (95% CI, 47.5% to 71.0%) for gestational ages less than 26 weeks and 32.1% (95% CI, 24.8% to 44.4%) for gestational ages 26 weeks or older (Table 2). We also analyzed the primary outcome by participating site and day after birth, gestational age group and day of age, and day of age (eFigures 5, 6, and 7, respectively, in Supplement 2).

**Table 2.** Primary Outcome by Participant Gestational Age

Gestational age, wk	Burden of outcome, median (IQR), % hours		*P* value	Relative change, % (95% CI)^a^
	Intervention group	Standard care group		
**23**^+0^ **to 25**^+6^				
Participants, No.	15	14	NA	NA
Cerebral hypoxia and hyperoxia	5.0 (2.9 to 13.0)	51.6 (38.5 to 96.4)	<.001	57.0 (47.5 to 71.0)
Cerebral hypoxia	1.1 (0.3 to 2.2)	5.3 (2.6 to 18.2)	.04	222.6 (82.9 to 487.5)
Cerebral hyperoxia	3.3 (2.3 to 10.9)	47.2 (23.3 to 87.3)	<.001	61.8 (50.0 to 81.1)
**26**^+0^ **to 28**^+6^				
Participants, No.	35	36	NA	NA
Cerebral hypoxia and hyperoxia	5.9 (2.5 to 14.7)	23.8 (5.8 to 67.0)	.07	32.1 (24.8 to 44.4)
Cerebral hypoxia	0.7 (0.04 to 1.6)	1.8 (1.0 to 7.1)	.009	465.1 (134.5 to 525.8)
Cerebral hyperoxia	4.5 (1.9 to 14.1)	13.4 (4.2 to 44.0)	.43	21.1 (13.9 to 40.8)

Abbreviation: NA, not applicable.

^a^ Model adjusted for gestation, chorioamnionitis, and birthweight percentile.

### Secondary Outcomes

No significant differences were observed between the 2 groups for secondary outcomes assessed before hospital discharge (Table 3). For example, the adjusted relative risk for mortality before home discharge was 1.28 (95% CI, 0.21-7.92; *P* = .78).

**Table 3.** Secondary Outcomes Before Hospital Discharge

Outcome	Participants, No. (%) (N = 100)		*P* value	aRR (95% CI)^a^
	Intervention group (n = 50)	Standard care group (n = 50)		
Mortality before home discharge	3 (6.0)	3 (6.0)	.78	1.28 (0.21-7.92)
Brain injury before hospital discharge	16 (32.0)	17 (34.0)	.58	0.77 (0.30-1.94)
Any intraventricular hemorrhage	16 (32.0)	16 (32.0)	.78	0.88 (0.35-2.20)
Severe intraventricular hemorrhage (grades III and IV)	3 (6.0)	2 (4.0)	.75	1.38 (0.18-12.94)
Cerebellar hemorrhage	0	2 (4.0)	.79	1.32 (0.14-12.68)
White matter injury	1 (2.0)	2 (4.0)	.36	0.27^b^
Chronic lung disease at 36 weeks’ postmenstrual age^c^	27/47 (57.0)	31/48 (65.0)	.83	0.91 (0.37-2.28)
Necrotizing enterocolitis ≥stage 2	3 (6.0)	3 (6.0)	.83	0.84 (0.16-4.38)
ROP	29 (58.0)	32 (64.0)	.94	0.97 (0.90-1.04)
Surgery for ROP	2/29 (7.0)	5/32 (16.0)	.25	0.35 (0.04-1.90)
Anti-VEGF therapy for ROP	3/29 (10.0)	6/32 (19.0)	.33	0.42 (0.06-2.31)

Abbreviations: aRR, adjusted relative risk; ROP, retinopathy of prematurity; VEGF, vascular endothelial growth factor.

^a^ Model adjusted for gestation, chorioamnionitis, and birthweight percentile.

^b^ Model did not converge.

^c^ Excludes infants who died before 36 weeks’ postmenstrual age.

### Safety

No participant in the intervention group developed skin injury from the NIRS sensor. One participant in the standard care group developed a transient imprint without skin injury from the NIRS sensor. The data safety committee did not identify any additional safety issues for the infants.

## Discussion

In this RCT, we demonstrated that treatment guided by cerebral oximetry compared with treatment based on conventional clinical monitoring and blinded cerebral oximetry significantly improved the stability of cerebral oxygenation during the first 5 days after birth in premature infants born at less than 29 weeks’ gestation. From a safety perspective, no infant in the intervention group experienced skin injury related to cerebral oximetry throughout the intervention period. In contrast, 1 infant in the standard care group developed a transient skin imprint from the NIRS sensor, which resolved after sensor repositioning. There were no significant differences between groups in key secondary outcomes related to major morbidities or mortality before hospital discharge. Although treatment rates for advanced ROP were lower in the intervention group, this difference was not significant. Overall, secondary outcomes were similar given that the study was not powered to detect modest differences in outcomes with low event rates, such as advanced ROP requiring treatment or mortality. These findings warrant further exploration in adequately powered trials, with consideration of gestational age and intervention duration.

The findings from the NIRTURE trial provide valuable insights into the stability of cerebral oxygenation in extremely preterm infants during the transitional phase. The SafeBoosC-II study^10^ previously demonstrated significantly improved stability of cerebral oxygenation from the intervention. In the SafeBoosC-II study,^10^ cerebral hypoxia but not cerebral hyperoxia contributed to the primary outcome. The improvement in primary outcome in the NIRTURE trial was predominantly driven by a reduction in cerebral hyperoxia, although a significant decrease in cerebral hypoxia was also observed.

Both NIRTURE and SafeBoosC-II trials demonstrated that the combination of cerebral oximetry and a dedicated treatment guideline improved cerebral oxygenation stability with no adverse effects from the intervention. It is important to acknowledge methodological differences between the 2 trials, which may influence the interpretation and generalizability of their findings. Compared with the SafeBoosC-II study, the NIRTURE trial’s methods included using a single NIRS device manufacturer with a neonatal population–specific sensor; an extended intervention period covering from 72 hours after birth to include the first 5 days after birth; real-time, unmodified device alarms for detecting cerebral hypoxia, hyperoxia, or both; elimination of device algorithm variability by using a NIRS device from 1 manufacturer; using 65% to 90% as the reference range for cerebral oxygenation; and having participating site investigators but few clinical staff with prior experience with cerebral oximetry. The rationale for the previously listed methods of the NIRTURE trial is detailed in Jani et al.^12^ While the SafeBoosC-II study demonstrated the stability of cerebral oxygenation from the intervention in the first 72 hours after birth, the NIRTURE trial demonstrated further extension of this finding to the first 5 days after birth. The absence of significant differences in major morbidities before hospital discharge suggests that the intervention is safe in this at-risk population.

Disturbances in cerebral perfusion and oxygenation, particularly during the early postnatal phase, are significant contributors to brain injury in preterm infants. These disruptions are associated with intraventricular and cerebellar hemorrhage, periventricular leukomalacia, and cerebral atrophy.^17^ Stabilizing cerebral perfusion and oxygenation may reduce this risk and improve neurodevelopmental outcomes. The SafeBoosC-III trial^18^ found that cerebral oximetry–guided treatment during the first 72 hours after birth did not reduce mortality or severe brain injury at 36 weeks’ postmenstrual age in extremely preterm infants. As highlighted by Chock et al,^19^ several limitations may have influenced these results, including the use of different NIRS monitors, reliance on cranial ultrasonography for brain injury detection, a short intervention period potentially missing later cerebral hypoxia, limited site experience with NIRS interpretation, and protocol compliance–monitoring issues. These challenges highlight the complexity of cerebral NIRS research and underscore the need for robust study design in future research. Such research could build evidence on cerebral oxygenation stability with treatment guided by changes in cerebral NIRS from baseline and automated control of oxygen delivery. Major strengths of our trial include using a commercially available, unmodified, single type of NIRS device with a neonatal-specific sensor for continuous cerebral oximetry; active involvement of parents with a lived experience of preterm birth who were integral to the study from conceptualization through development; minimizing bias by blinding data analysts to treatment allocation; multisite recruitment and 2 consenting approaches that facilitated the inclusion of infants born after hours and under emergency conditions, thus improving generalizability of the findings to broader settings and population; comprehensive training of all clinical staff in cerebral oximetry at participating sites, reflecting real-world effective education strategy; including deviations in cerebral oxygenation lasting more than 1 minute in the analysis, which balanced the risk of acting on false alarms from poor sensor contact with the participant and identifying true real-time deviations; and a low risk of attrition bias, with 4 infants excluded from the analysis after randomization.

### Limitations

This study has several limitations. Multiple births beyond twins were excluded due to limited numbers of available NIRS devices required to deliver the intervention. However, no such births occurred at participating sites, minimizing the practical impact of this exclusion criterion. There is a potential for inadvertent selection bias given that a higher proportion of infants in the intervention group were exposed to chorioamnionitis. These imbalances were addressed through statistical adjustment in the analysis of the primary outcome. Interestingly, despite exposure to chorioamnionitis, often associated with respiratory instability, the intervention improved stability of cerebral oxygenation. The study had no infants at less than 23 weeks’ gestation; therefore, the benefit of the intervention in these infants needs exploration. In the absence of international guidelines on handling NIRS artifacts, the artifacts were included in the data analysis.

## Conclusions

In this RCT of cerebral oxygenation, cerebral oximetry–guided treatment significantly improved stability of cerebral oxygenation in extremely preterm infants during the first 5 days after birth. Neurodevelopmental outcomes for infants enrolled in this trial are still being collected, and they will be reported later. Whether improved cerebral oxygenation translates into better neurological outcomes in early childhood remains uncertain.^20^ Resolving this uncertainty will require well-conducted RCTs and an individual-patient data meta-analysis.^21^ Such trials should incorporate alternate consenting approaches, such as a waiver or deferred consent, in accordance with regional regulatory and ethical compliance.^22^

## Notes

Supplement 1.
Trial Protocol and Statistical Analysis Plan

Supplement 2. eFigure 1. Clinical treatment algorithm for cerebral hypoxia (crSO2 <65%) eFigure 2. Clinical algorithm for cerebral hyperoxia (CrSO2 >90%) eFigure 3. Interventions for cerebral hypoxia eFigure 4. Interventions for cerebral hyperoxia eTable 1. Primary outcome using 10-min trigger interval stratified by gestational age of participants eTable 2. Treatment and complications in participants within the first 5 d after birth eFigure 5. Primary outcome by participating site and day after birth eFigure 6. Primary outcome by gestational age groups and day of age after birth eFigure 7. Primary outcome by day of age after birth

Supplement 3.
Data Sharing Statement
