# Supplementary material for: Cerebral Oximetry–Guided Treatment and Cerebral Oxygenation in Extremely Preterm Infants: A Randomized Clinical Trial
Source: JAMA Netw Open. 2026 Feb 5;9(2):e2557620. doi: 10.1001/jamanetworkopen.2025.57620 (PMC12878427; doi:10.1001/jamanetworkopen.2025.57620)
Supplement: Supplement 1. — Trial Protocol and Statistical Analysis Plan [file jamanetwopen-e2557620-s001.pdf]

Non-Drug/Device Protocol Template

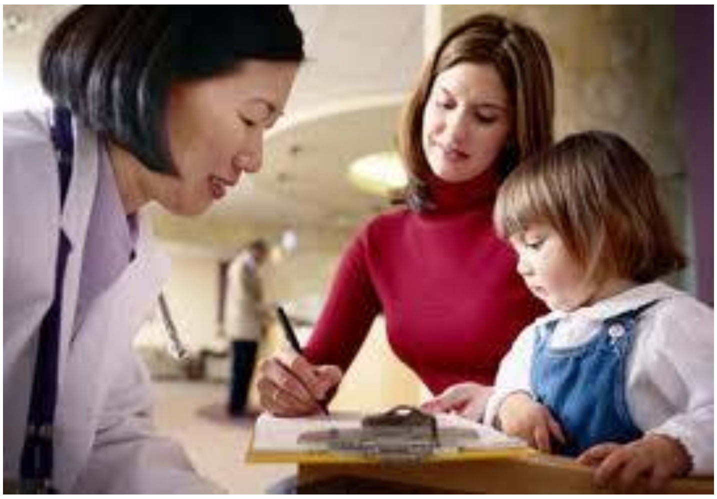

Published Date: August 2013

Review Date: August 2014

NOTES TO USERS

**Who should use this template?**	Anyone conducting clinical research which does **not** involve drugs or devices.
**Why do you need a protocol?**	The protocol is essential for study conduct, review, reporting, and interpretation.
**Why use this template?**	This non-drug template has been modified from the SPRIRIT (**S**tandard **P**rotocol **I**tems: **R**ecommendations for **I**nterventional **T**rials). The Spirit Statement is an international initiative that aims to improve the quality of clinical trial protocols by defining an evidence-based set of items to address in a protocol.<br><br>Reference: Chan et al., (2013) SPIRIT 2013 Explanation and Elaboration: Guidance for protocols of clinical trials. *BMJ 2013; 346:e7586*
**How do I use this template?**	There is a brief explanation under each heading stating the information that should be contained in that section.<br><br>You will need to input your study specific information under each heading and remove explanatory information.<br><br>As this is a template, users are remaindered that not all examples may be applicable to their study. Please contact your institution to discuss specific protocol questions.
**Do I still need to complete the National Ethics Application Form (NEAF)**	Yes – you must finalise your protocol prior to completing the NEAF. The NEAF is a form used by ethics committees to conduct standard review of all projects. While you need to refer to your protocol to answer most questions in the NEAF, it does not replace the need for a detailed protocol.
**Copyright**	This template is licensed under the Creative Commons Attribution NonCommercial-NoDerivs 3.0 Unported License

PROTOCOL

Targeted cerebral oxygenation using dedicated treatment versus usual care in extremely preterm infants: protocol for a multicentre international phase II randomised controlled trial

[**N**ear **I**nfra-**R**ed spectroscopy **T**argeted **U**se to **R**educe adverse outcomes in **E**xtremely preterm infants (NIRTURE trial).]

Protocol Number (if applicable):
Version: 1.12
Date: 14/12/2022

Author/s:

Sponsor/s:
None (finding application made, outcome is pending)

CONFIDENTIAL

This document is confidential and the property of Westmead Hospital. No part of it may be transmitted, reproduced, published, or used without prior written authorization from the institution.

Statement of Compliance

This document is a protocol for a research project. This study will be conducted in compliance with all stipulation of this protocol, the conditions of the ethics committee approval, the NHMRC National Statement on ethical Conduct in Human Research (2007) and the Note for Guidance on Good Clinical Practice (CPMP/ICH-135/95).

Table of Contents

Contents

Table of Contents 4

1. Glossary of Abbreviations & Terms 6

2. Study Sites 6

2.1 Study Location/s 6

3. Funding and Resources 6

3.1 Source/s of Funding 7

4. Introduction/Background Information 7

4.1 Lay Summary 7

4.2 Introduction 7

4.3 Background information 7

5. Study Objectives 10

5.1 Research Question 10

5.2 Primary Objectives 10

5.3 Secondary Objectives 10

5.4 Outcome Measures 10

6. Study Design 11

6.1 Study Design Diagram 11

6.2 Study Type & Design & Schedule 12

6.3 Standard Care and Additional to Standard Care Procedures 17

6.4 Randomisation 18

6.5 Study methodology 18

7. Study Population 22

7.1 Recruitment Procedure 22

7.2 Inclusion Criteria 22

7.3 Exclusion Criteria 22

7.4 Consent 23

8. Participant Safety and Withdrawal ... 23
8.1 Risk Management and Safety ... 23
8.2 Adverse Event Reporting ... 23
8.3 Handling of Withdrawals ... 25
8.4 Replacements ... 25
9. Statistical Methods ... 25
9.1 Sample Size Estimation & Justification ... 25
9.2 Power Calculations ... 25
9.3 Statistical Methods To Be Undertaken ... 25
10. Storage of Blood and Tissue Samples ... 26
10.1 Details of where samples will be stored, and the type of consent for future use of samples ... 26
11. Data Security & Handling ... 26
11.1 Details of where records will be kept & How long will they be stored ... 26
11.2 Confidentiality and Security ... 26
11.3 Ancillary data ... 27
12. Appendix ... 27
13. References ... 27

1. GLOSSARY OF ABBREVIATIONS & TERMS

Abbreviation	Description (using lay language)
CI	Chief investigator
PI	Principal investigator
NICU	Neonatal intensive care unit
CP	Cerebral palsy
NIRS	Near Infra-Red Spectroscopy
RR	Relative risk
CI	Confidence interval
RCT	Randomised controlled trial
PMA	Post menstrual age
MRI	Magnetic resonance imaging
GMA	General Movements assessment
HINE	The Hammersmith Infant Neurological Examination
TIMP	The Test of Infant Motor Performance
GMFCS	Gross Motor Functional Classification System
SD	Standard deviation
CLD	Chronic lung disease
SpO_2	Pulse oximetry
HR	Heart rate
TMC	Trial management committee
DSMC	Data safety and management committee

2. STUDY SITES

.

2.1 STUDY LOCATION/S

Three tertiary NICUs in Australia, 1 in New Zealand and 1 in the US.

3. FUNDING AND RESOURCES

3.1 Source/s of Funding

This is an investigator-initiated research. Application has been made for a competitive research grant to fund the essential equipment, research nurse and clinical trial coordinator.

4. Introduction/Background Information

4.1 Lay Summary

Preterm babies can develop brain injury which is associated with long-term developmental problems. Most brain injuries occur within the first 5 days of life and protection of the brain during this critical period is essential in preventing brain damage. Fluctuating brain oxygen levels is one reason for brain injury to develop, which can also cause bleeding in the brain. Keeping brain oxygen levels in a specific range may be protective. The purpose of the study is to investigate in preterm babies, whether it is possible to keep brain oxygen levels in a specific range for the first 5 days by NIRS monitoring and following a dedicated clinical treatment guideline. Babies in this study will have either (a) the usual treatment (given to all babies), or (b) they will be in the group where the focus is on keeping brain oxygen levels in a specific range for the first 5 days of life using NIRS monitoring.

4.2 Introduction

Premature babies are a group of babies with high risk of complications including brain injury and death. This risk of developing brain injury or dying is high in the first few days after birth. Near infrared spectroscopy is a bedside non-invasive way to monitor the brain oxygen levels. There are no studies investigating the effects of targeted cerebral oxygen monitoring on mortality before hospital discharge and long-term neurodevelopment. Prior to undertaking a large multi-site randomized control trial, we are conducting a phase II study.

NIRTURE study aims to address the following research question: In premature babies (born before <29 weeks gestation), can a dedicated treatment guideline combined with cerebral oxygenation monitoring (intervention group) compared to usual treatment with blinded cerebral oxygenation monitoring (control group) reduce the burden of cerebral hypoxia and hyperoxia in the first 5 days?

The proposed study will address the limitations that affects the generalizability of findings from SafeBoosC II study (details below in section 4.3) and extend our understanding on whether the combination of cerebral oxygenation monitoring and a dedicated treatment guideline will reduce the burden of cerebral hypoxia and hyperoxia?

4.3 Background information

Background and introduction:

Premature babies are very fragile and vulnerable, especially those born less than 29 weeks gestation. They are at a greater risk of death, cerebral palsy, intellectual impairment, learning disorders, vision and/or hearing loss (1-3). In 2017, approximately 1600 premature infants < 29 weeks gestation age were born and admitted to neonatal intensive care units in Australia and New Zealand. This is a vulnerable, high risk group with a combined adverse outcome incidence of ~33% (for rates of mortality and moderate to severe functional impairments) (4).

The risk of cerebral palsy is high in extremely preterm babies (12% vs 1% in term born) (5). There are significant expenses to the family and associated economic impact in caring for a child with cerebral palsy, estimated at $43,431 per annum and up to $115,000 if loss of wellbeing is

considered (6). The mechanism of brain injury in extremely preterm infants is multi-factorial. One proposed mechanism is the immature brain is exposed to periods of low oxygen (cerebral hypoxia) and/or periods of high oxygen (cerebral hyperoxia).

Large multi-site randomised controlled trials comparing lower (85-89%) versus higher (91-95%) peripheral saturation target ranges in extremely preterm infants found increased risk of mortality and neonatal morbidities for infants outside target range, but the trials did not identify differences in neurodevelopmental outcomes, including cerebral palsy (7-13). Cerebral near infra-red spectroscopy (NIRS) monitoring is a safe, non-invasive optical technology that allows assessment of tissue oxygenation of the brain (14), thus providing a non-invasive measure of the blood flow to the brain.

Recently, investigators from the SafeBoosC study completed a phase II trial (15,16) where 166 extremely preterm infants were randomised to cerebral oxygenation monitoring combined with a dedicated clinical treatment guideline (experimental group) versus blinded NIRS monitoring and treatment as usual (control group). This trial found that NIRS monitoring in combination with a dedicated clinical treatment guideline successfully reduced the burden of hypoxia and hyperoxia from 81% hours to 36% hours during the first three days of life (p<0.001). Furthermore, the proportion of severe brain injury at term age assessed by cranial ultrasound was 12.5% in the experimental group versus 23.4% in the control group, (RR 0.53, 95% CI: 0.26 to 1.08). Mortality was 14% in the experimental versus 25% in the control group-(RR 0.50 (95% CI: 0.29 to 1.00), this study was under-powered for developmental outcomes as shown in Table 1.

However, this study had certain limitations that affects the generalisability of the findings:

1. Use of 4 different NIRS devices with an adult sensor: There are systematic differences in the measurement with different devices and different sensors (adult versus neonatal sensors) (15, 16). Studies using adult sensors have reported 55%-85% as the normal range for cerebral tissue oxygenation (17). When using newborn sensors, the lower range of normal has been reported as 65% (mean difference of 10%). Where an adult sensor is used, a correction factor needs to be applied to generate measurements equivalent to newborn sensors. In our study, we chose the range of 65% - 90% based on review of literature and local data from monitoring of preterm infants at Westmead Hospital's NICU.
2. Changing the intrinsic alarm mechanism: use of a "burden alarm" that warned when hypoxia or hyperoxia was accumulating. This is in contrast to real world situation where the device alarms within 1.5 seconds of deviation from the target range.
3. Use of an aggressive dedicated clinical treatment guideline in SafeBoosC II study. For example, as per SafeBoosC II study: 'if the cerebral oxygenation is below 55%, then assess cardiovascular status. If Blood pressure below the normal range or low, **even in the normal range**, consider Vasopressor-inotropes, Fluid bolus (normal saline), Decrease mean airway pressure'.

In summary, SafeBoosC II study found a significant reduction in the burden of cerebral hypoxia and hyperoxia in the group that were randomised to cerebral oxygenation monitoring combined with a dedicated clinical treatment guideline (experimental group) versus the group with blinded NIRS monitoring and treatment as usual (control group). Could this finding be explained by aggressive clinical approach in experimental group, and /or heterogeneity in the equipment used, this remains unknown. It is also important to note that NIRS offers a role in monitoring regional tissue oxygenation.

In our study, we are addressing weaknesses from SafeBoocS II study by:

(i) eliminating equipment heterogeneity (by using same NIRS equipment and non-adhesive neonatal NIRS sensor),
(ii) by using non-adhesive sensors, the risk of skin injury is extremely low and
(iii) less aggressive clinical guideline to ensure harm from unnecessary treatment is reduced and
(iv) inclusion of long-term neuro-developmental follow up.

We believe that prior to conducting a large multisite international RCT investigating the impact of using a combination of cerebral NIRS monitoring and dedicated clinical treatment guideline on composite outcome of death before hospital discharge and neuro-developmental outcome at 2 years corrected age, it is important to conduct a phase II RCT incorporating important differences to SafeBoosC – II study. It is important to investigate if a significant difference truly exists by following a dedicated conservative clinical guideline combined with cerebral oxygenation monitoring (intervention group) versus treatment as usual and blinded cerebral oxygenation monitoring (control group).

Table 1 Data from previous RCT of cerebral oxygen targeting in preterm infants

Outcome	NIRS guided algorithm	Placebo	Risk Ratio (95%CI)	P value (un adjusted)	P value (adjusted)
**Median (IQR) burden of hypoxia and hyperoxia (% hours)**	36.1 (9.2-79.5)	81.3 (38.5-181.3)	-58 (-35 to -74)		< 0.001
**All-cause mortality, n (%)**	12/86 (14)	20/80 (25)	0.50 (0.29 to 1.00)	0.049	0.10
**Brain Injury on US, n (%)**				0.053	0.11
None	21/80 (26)	26/77 (34)			
Mild-Moderate	49/80 (61)	33/77 (43)			
Severe	10/80 (13)	18/77 (23)			
**Necrotising enterocolitis, n (%)**	9/86 (11)	10/80 (13)	0.83 (0.33 to 1.94)		0.69
**Bronchopulmonary Dysplasia, n (%)**	41/72 (57)	28/60 (47)	1.27 (0.94 to 1.50)		0.20
**Retinopathy of Prematurity, n (%)**	14/86 (16)	8/80 (10)	1.64 (0.75 to 3.00)		0.20

Currently, SafeBoosC III study is ongoing which is a large, pragmatic trial in extremely preterm babies to examine if cerebral oximetry combined with a treatment guideline can reduce the risk of death or severe brain injury at 36 weeks of age. (https://www.rigshospitalet.dk/english/departments/juliane-marie-centre/department-of-neonatology/research/SafeboosC-III/Sider/default.aspx).

There are no adequately powered RCT's investigating stability of cerebral oxygenation and impact on neurodevelopmental outcomes (until 5 years of age). While it is very encouraging that CP rates for preterm infants have recently declined in Australia, there is still much to do in preventing brain injury in extremely preterm infants especially preventing brain injury at birth and in the first few days of life. Evidence supports hypoxia contributing to mortality as well as brain injury.

There is an urgent need for a safe and effective non-invasive clinical approach incorporating cerebral NIRS monitoring, with good evidence to support its use(18). However, there are no studies of this nature, investigating the effects of targeted cerebral oxygen monitoring on mortality before hospital discharge and long-term neurodevelopment.

Additionally, it is important to note, in Australia and New Zealand, the standard practice for all premature babies who are born before 29 weeks gestation, is to have the following:

- Serial cranial ultrasounds
- MRI brain if clinical concerns or cranial ultrasound evidence of brain injury
- Regular neuro-developmental assessments till 5 years of age.

5. Study Objectives

5.1 Research Question

In premature babies (<29 weeks gestation at birth), can using a dedicated treatment using cerebral oxygenation monitoring compared to usual treatment reduce the burden of cerebral hypoxia and hyperoxia in the first 5 days?

5.2 Primary Objectives

In premature babies (< 29 weeks gestation at birth), can a dedicated treatment with cerebral oxygenation monitoring compared to usual treatment, reduce the burden of cerebral hypoxia and hyperoxia in the first 5 days?

We hypothesise that the burden of hypoxia and hyperoxia can be reduced (by half) by a combination of specific treatment guideline and keeping cerebral oxygenation in the specific range of 65-90%.

5.3 Secondary Objectives

To explore the relationship between cerebral hypoxia and hyperoxia in the first 5 days and the following:

- Mortality before hospital discharge.
- Brain injury before hospital discharge.
- Neonatal morbidities (chronic lung disease, necrotizing enterocolitis, retinopathy of prematurity) before hospital discharge.
- Neuro-developmental outcomes until 5 years of age.
- Physiological functions (heart rate, pulse oximetry, blood pressure) in the first 5 days.
- Physiological functions (observation of pulse oximetry) at 36-37 weeks PMA.
- Sleep architecture (based on sleep study) at 36-37 weeks PMA (at Westmead and Wellington sites only) including cerebral NIRS monitoring.

5.4 Outcome Measures

Primary outcome:
The burden of cerebral hypoxia and hyperoxia during the first 5 days expressed as percent hours.

Exploratory secondary outcomes:

- Newborn mortality rates prior to home discharge
- Brain injury based on cerebral imaging prior to home discharge
- Neonatal morbidities (chronic lung disease, necrotizing enterocolitis, retinopathy of prematurity) prior to home discharge
- Physiological functions (observation of pulse oximetry) at 36-37 weeks PMA.
- Sleep architecture (based on sleep study) at 36-37 weeks PMA (at Westmead and Wellington sites only) including cerebral NIRS monitoring.
- Motor performance assessment (General movements assessment) during NICU stay and at 4 months corrected age follow up.
- Neuro-developmental outcomes until 5 years of age.
- Measurement of physiological functions (heart rate, pulse oximetry, blood pressure) in the first 5 days.

6. Study Design

6.1 Study Design Diagram

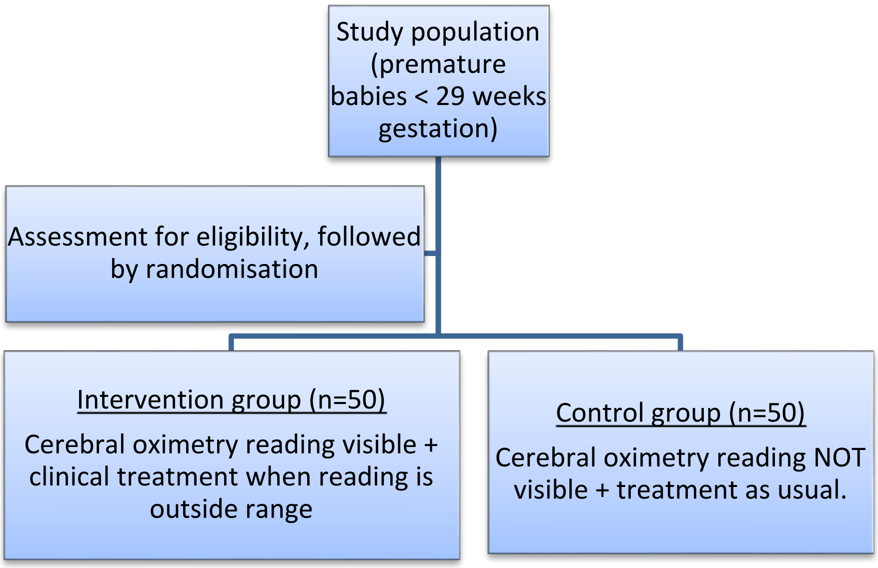

CONSORT 2010 Flow Diagram

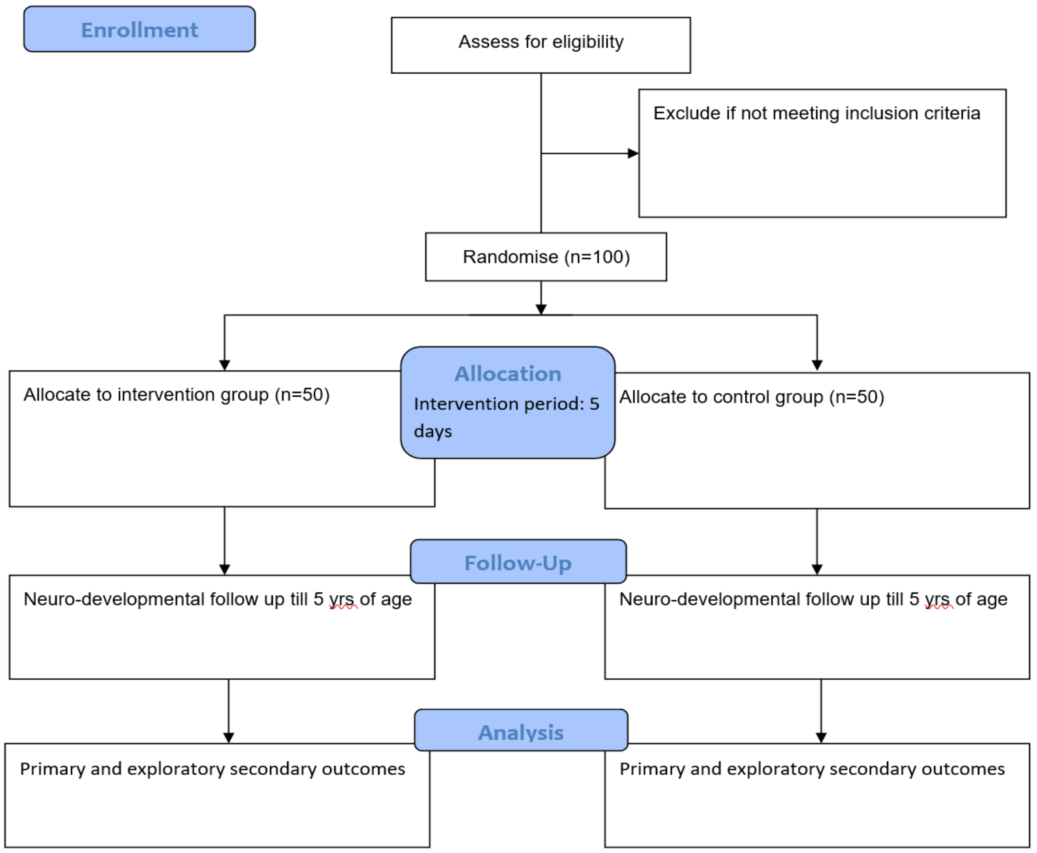

6.2 Study Type & Design & Schedule

1. This trial will be a single-blinded randomised controlled trial with two parallel groups that are stratified for site and gestational age (<26 weeks and ≥ 26^{+0} – 28^{+6} weeks gestation).

2. <u>Inclusion criteria:</u>

   - Preterm infants (singleton or twin births) <29 weeks gestation who are either inborn (born at the local study site hospital) or outborn (born outside the local study site hospital) and less than 6 hours of age when admitted to the study site NICU.

   <u>Exclusion criteria:</u>

   - Infants admitted to NICU beyond 6 hours of age.
   - Infants with an antenatal or postnatal diagnosis of major congenital anomaly requiring major surgery or a genetic disorder associated with neurological impairment.
   - Multiple births beyond twins are excluded for practical reasons (sites may not have additional NIRS monitors and the rates of multiple births beyond twins in Australia and New Zealand is very low < 2%).

3. This trial will be a multi-site, international study. Participating sites will be NICUs skilled with provision of tertiary level of intensive care services to preterm infants born < 29 weeks gestation.

4. The study design will assist in assessment of the primary outcome that is ‘in premature babies (< 29 weeks gestation at birth), can dedicated treatment using cerebral oximetry compared to usual treatment, reduce the burden of cerebral hypoxia and hyperoxia in the first 5 days”? A RCT will detect any difference in hypoxia and hyperoxia burden between groups (primary outcome) and allow exploration of secondary outcomes.

5. The following data will be collected:

A. Cerebral oxygenation and physiological data:

- NIRS device collects data in real time at 1.5 seconds interval. This data is stored in the memory drive of the SenSmart device. Data from each patient will be downloaded using a SenSmart data management software (this software is available at each site for the study) after the completion of the monitoring period (5 days). This data will be stored in CSV format. This data will be read by local PI and then by the central reporting team at Westmead Hospital. Data will be transferred using secure work-based email or Accellion App or secure transfer platforms as approved by the local health authorities. File will be coded by the local PI; central reporting team will have de-identified version of the file.
- There is a plan for an upgrade of NIRS monitors to have neonatal pulse oximetry incorporated which will collect data on pulse oximetry (SpO_2) and heart rate (HR). If this upgrade is available at the time of commencement of the study, data for SpO_2 and HR will be downloaded simultaneously using the previously mentioned software. If this is not available, then we will collect data on SpO_2 and HR from a Masimo devices or a similar pulse oximetry device (which are used routinely in NICUs to monitor pulse oximetry). The clocks on both devices (NIRS and Masimo) will be synchronised13 prior to commencement of monitoring for every patient that is enrolled. Data from Masimo pulse oximeters will be downloaded using a software program into CSV format. This data will be read by local PI and then by the central reporting team at Westmead Hospital. Data will be transferred using secure work-based email or Accellion App or secure file transfer platforms as approved by the local health authorities. File will be coded by the local PI; central reporting team will have de-identified version of the file. ICM+ software program will be used at 4 sites (US, New Zealand, Nepean and RHW) for centralised physiological data collection from the NIRS and the bedside clinical monitors. Westmead site has a similar software program (iXcellence) already in clinical use. Both these softwares offer identical data collection and ability to transfer data to CSV format for ease of statistical analysis. For the US site, NONIN NIRS monitors will be used for collection of primary outcome data. Other physiological data (for some aspects of secondary analysis) will be obtained when technical issues with ICM+ installation and connectivity have resolved.
- Other physiological functions (blood pressure): This data will be collected from medical records and the response to alarm form. File will be coded by the local PI; central reporting team will have de-identified version of the file.

B. Cerebral imaging (cranial ultrasound and MRI brain): Cranial ultrasounds are routinely performed for all premature babies at regular intervals. All ultrasounds and MRI brain are routinely reported by an independent radiologist. De-identified images will be transferred by the local PI to the central reporting team (Neuro-radiologists KP and RG) using an electronic file transfer system approved by local health authority.

C. Data on mortality, and neonatal morbidities is routinely collected for all premature babies. An electronic data sheet will be generated, each site PI will code the patient and maintain local data. This will be transferred to the Central reporting team by an electronic data transfer platform as approved by the local health authorities. The central reporting team will collate the

de-identified data for all patients. This will be password protected file and access will be only be to local PI (for local data) and central reporting team (for all patients). For babies transferred to other hospitals, data on outcomes will be collected to ensure completeness of data.

D. All study participants will undergo a oneoff data download of their physiological functions (pulse oximetry for approximately 12-24 hours) at 36-37 weeks PMA. Local PI will maintain a data record and de-identified data will be transferred to central CI team. Please note, pulse oximetry is standard/routine monitoring for all babies in NICU.

E. Study participants at Westmead and Wellington will undergo a one-off sleep study during the day at 36-37 weeks PMA to evaluate their sleep architecture including cerebral NIRS monitoring. Local PI will maintain a data record and de-identified data will be transferred to central CI team.

F. Neuro-developmental outcomes: Premature babies routinely undergo neuro-developmental assessment at regular intervals until 5 years of age. This data will be collected using the above-mentioned electronic data sheet as described for point C.

General movements (motor performance) assessment using a short video is a part of routine neuro-developmental assessment. This is accessed by recording a short video (2-3 minutes). Local PI will maintain record of this video (coded) and transfer using an electronic file transfer system approved by local health authority. After the central reporting team has performed motor performance reporting, the video will be deleted by the central reporting team. Local PI will continue to retain a copy for medical records. All sites have a high-risk neuro-developmental follow up clinic with staff trained in performing GM's, Bayley Scales of Infant and Toddler development and Wechsler Preschool and Primary Scale of Intelligence at 5 years of age. All participating centres are using the same assessment tools at follow up.

6. REDCap will maintain a list of participants enrolled. Each site PI will access only to their site records. The chief investigators and clinical trials coordinator (if funding available) will have access to all sites to oversee enrollment. Participants will be given unique codes to protect their identity on all documentation utilised14 for recording and analysis purposes. Separate data records will be maintained for identifiable and de-identified data. Site PI will be responsible for collating and maintaining local site data. Other data storage and handling as described in point 5 above. Data will be stored for a minimum of 15 years or until the youngest participant turns 25 (whichever is the longest).

Time frame for each component of the study is as follows:

Component of the study	Time frame	Analysis period
NIRS and physiological functions	For first 5 days of life	Before discharge
Physiological functions	Data download of overnight pulse oximetry once (for approximately for 12-24 hours) at 36-37 weeks PMA	Before discharge
Sleep architecture	Day sleep study once (for approximately for 6 hours) at 36-37 weeks PMA	Before discharge
Cranial imaging	Prior to home discharge	4 months post discharge
Neonatal mortality and morbidities	Prior to home discharge	4 months post discharge
Neurodevelopmental follow up	Until 5 years of age	After completion of 5 years assessment.

7. There are no home visits.

8. Ensure you have included all information on all required contingency plans within your study outline. Yes.

9. No, this study will not be used for student project.

10. Flowchart or table specifying visits, interventions and other relevant details

			STUDY PERIOD								
**TIMEPOINT**	**Enrolment**	**Allocation**	**T1**	**T2**	**T3**	**T4**	**T5**	**T6**	**T7**	**T8**	**T9**
ENROLMENT:											
Eligibility screening	**X**										
Informed consent	**X**										
Allocation		**X**									
INTERVENTION:											
Intervention group			**X**								
Control group			**X**								
ASSESSMENTS:											
Skin Integrity Tool			**X**								
Record of response to alarm			**X**								
Cranial ultrasounds				**X**	**X**						
Data download of overnight pulse oximetry (once)						**X**					
Day sleep study (once)						**X**					
MRI brain (where performed)							**X**				
Motor performance						**X**		**X**			
Neuro-developmental follow up								**X**	**X**	**X**	**X**

T1 =Post-allocation to 5 days from birth; **T2** = first week from birth; **T3** = 32 weeks PMA or 6 weeks after birth; **T4** = 36-37 weeks PMA; **T5** = term PMA (37-40 weeks); **T6** = Follow-up assessment at 3-4 months corrected age, T7 = 12 months corrected age, T8 = 18-24 months corrected age, T9 = 5 years of age.

6.3 STANDARD CARE AND ADDITIONAL TO STANDARD CARE PROCEDURES

Standard Care Procedures		
**Procedure**	**Time/Visit**	
Skin assessment	Entire duration of admission	
Cranial imaging	At regular intervals over the duration of admission	
Physiological function monitoring (pulse oximetry, recording heart rate, blood pressure)	Heart rate, pulse oximetry is recorded continuously, blood pressure intermittently using blood pressure cuffs or invasively if there is an arterial catheter when clinically indicated	
Mortality and morbidity data	Entire duration of admission	
Motor performance	At 32 week and 36-37	

Additional To Standard Care	
**Procedure**	**Time/Visit**
NIRS monitoring	First 5 days (for intervention) and at 36-37 weeks PMA (for observation only with sleep study)
Day sleep study (once)	At 36-37 weeks PMA.

assessment	weeks PMA
Neuro-developmental assessment	At regular intervals until 5 years after discharge.

6.4 RANDOMISATION

Central randomisation can be performed by the investigator or by a delegate or by the medical team member (from NICU). Infants assessed as eligible will be randomised18 to control group or intervention group using variable block randomisation stratified for gestation age (< 26 weeks and ≥ 26 weeks) and study site using web based secure software (REDCap). Randomisation codes for the study will be generated by biostatistician (Liz Barnes has generated the codes). Permuted blocks will maintain group balance, infants will be allocated to two groups in a 1:1 ratio. Multiple births (twins) will be assigned to the same arm of the treatment (either intervention or control). Multiple births (twins) will be 18randomised to the same group, based on negligible intra-class coefficient (ICC) burden of hypoxia within pairs of twins in SafeBoosC-II study (16) and survey of parents of multiple births (19).

To ensure concealment, the block sizes will not be disclosed. Additionally, allocation concealment will be ensured, as REDCap will not reveal the randomisation until the infant has been recruited into the trial.

REDCap will maintain a list of participants enrolled. Each site PI will access only to their site records. The chief investigators will have access to all sites to oversee enrollment. Participants will be given unique codes to protect their identity on all documentation utilised18 for recording and analysis purposes. Separate data records will be maintained for re-identifiable and de-identified data.

Blinding (masking)

During intervention period: Due to the nature of the study, the assignment of infants to control arm or intervention arm cannot be blinded to the clinical staff (care providers). For infants in the control arm, the monitor screen will be blinded to the clinical team, this will be performed using an opaque cover over the monitor screen. For each monitor, it is possible to silence the low and high alarm, but keep the alarm ON for displacement of the sensors when the sensors are not making contact with the skin. Alarms for the range of cerebral tissue oxygenation (65-90) will be turned off and alarms for displacement/dislodgement of the sensors will remain active. Clinical staff (bedside nurse) will be provided with data collection sheet to determine the frequency of alarms and sensor repositioning during each shift for both arms.

Post-intervention period: Data analysts, other site personnel/investigator, outcome assessors and manuscript writers will be blinded to the intervention.

6.5 STUDY METHODOLOGY

Enrolment will occur after birth of the baby. After enrolment, infants will be randomised18 to either control group or intervention group. For all enrolled babies, monitoring of cerebral oxygenation will be commenced by placement of a non-adhesive probe on the fronto-parietal region of the forehead within 6 hours of birth and monitoring will be continued for 5 days.

For the Intervention group:

For the intervention group, the cerebral oxygenation reading is visible and the baby will be treated according to a dedicated clinical guideline when the cerebral oxygenation is outside the range of 65%-90% as shown in Figure 1 and Figure 2. The sensor site will be inspected based on the unit's practice of skin assessment to ensure correct sensor placement and skin integrity.

Clinical algorithm for cerebral hypoxia triggered when cerebral oxygenation ($CrSO_2$) is < 65% (Figure 1)

When Cerebral oxygenation ($CrSO_2$) is < 65% potential causes could include inaccurate sensor placement, low arterial oxygen saturation (20-23), reduced cerebral blood flow (23-38) and/or reduced haemoglobin concentration in circulating blood (39-43).

Figure 1: Clinical algorithm for cerebral tissue hypoxia

Important causes of cerebral hypoxia (low $CrSO_2$) include (but not limited to) conditions that decrease O_2 delivery to the brain such as low SpO_2, systemic hypotension, haemodynamically significant ductus arteriosus, hypocarbia and/or anaemia).

If no change to cerebral $CrSO_2$ from sensor repositioning, and $CrSO_2$ is consistently < 65% then check for the following (perform one change at a time and reassess within 30 minutes of making the change)

- Low peripheral oxygen saturation (SpO_2): if below normal or in the low normal range then aim to bring it back to normal range by performing maneuvers such as increasing FiO_2 and /or mean airway pressure.
- Abnormal cardiovascular status: presence of poor systemic perfusion and /or haemodynamically significant patent ductus arteriosus.
  - ✓ if clinical (capillary refill time ≥ 3 seconds or hypotension on non-invasive or invasive blood pressure measurement), or
  - ✓ biochemical (elevated serum lactate) or
  - ✓ echocardiographic signs (low cardiac output and/or low SVC flows) of poor systemic perfusion then consider administering a bolus of normal saline (10ml/kg) and /or inotropes.
  - ✓ Presence of haemodynamically significant patent ductus arteriosus (based on echocardiogram), consider medical treatment.
- Low PCO_2 level (transcutaneous or capillary/arterial blood gas): if below normal or low normal range, then aim to normalize by adjusting the mean airway pressure and / or minute ventilation.
- Low haemoglobin level: if the most recent Hb is low consider packed red cell transfusion.

If any of the standard parameters such as blood pressure, serum lactate, SpO_2 are deranged, clinician should target their correction despite $CrSO_2$ levels (even if they are in the normal range).

<u>Clinical algorithm for cerebral hyperoxia triggered when cerebral oxygenation (</u>$CrSO_2$) is > 90% (Figure 2)

When cerebral oxygenation ($CrSO_2$) is > 90% potential causes could include inaccurate sensor placement, high arterial oxygen saturation (9-12, 20, 21, 44-47), increased cerebral blood flow (24, 25, 35, 38) , and/or hypoglycaemia (48, 49).

<u>Figure 2: Clinical algorithm for cerebral hyperoxia</u>

Important causes of cerebral hyperoxia (high $CrSO_2$) include (but not limited to) conditions that increase O_2 delivery to the brain such as high SpO_2, hypercarbia, systemic hypertension, and/or hypoglycemia.

If no change to cerebral $CrSO_2$ from sensor repositioning, and $CrSO_2$ is consistently >90% then check for the following (<u>perform one change at a time and reassess within 30 minutes of making the change</u>)

- <u>High SpO_2</u>: If above normal or in the high normal range, aim to bring it back to normal range by performing manoeuvres such as decreasing FiO2 and / or MAP.
- <u>High PCO_2 level</u> (transcutaneous or capillary/arterial blood gas): if above normal or high normal range, then aim to normalize by adjusting the mean airway pressure and / or minute ventilation.
- <u>Abnormal cardiovascular status:</u> if hypertensive and receiving inotropes, considering weaning and/or ceasing inotropes.
- <u>Hypoglycemia:</u> if blood glucose level < 2.6 mmol/L, then increase glucose intake to maintain normal eu-glycaemia.

If no cause for high cerebral rSO_2 identified, then continue close clinical assessment.

Control group

For the Control group, cerebral oxygenation reading is NOT visible and the baby will be treated according to standard clinical practice. We will perform blinded NIRS monitoring (we will cover the monitor) and the information will not be available to clinicians to act upon.

Modifications

Intervention will be discontinued if requested by parents.

Adherence

In response to an alarm (for intervention and control group), an event will be marked by the bedside clinical team on the NIRS monitor. From the Response To Alarm form, NIRS data and medical records observation chart, we will ascertain adherence to the study algorithm and

actions performed. Research nurse/local PI will conduct compliance audit for both groups during the study.

For the intervention group: A bedside “Response to Alarm” form will be made available for staff to prospectively record actions performed for low &/ high cerebral oxygenation alarms during their shift to ensure compliance to the clinical algorithm as well as event mark in response to the alarm on the NIRS monitor.

Nursing and medical staff will receive education prior to study commencement on the application of NIRS sensors, skin care during monitoring period, measures to consider when there is cerebral hypoxia and/or cerebral hyperoxia. Bedside flowcharts will be available for staff to perform changes to clinical management. A laminated chart will be placed on the crib to remind staff of NIRS target range as well as a flow chart suggesting possible reasons for cerebral hypoxia/hyperoxia and suggested interventions.

For the control group: A bedside “Response to alarm” form will be made available for staff to record prospectively actions performed for sensor contact alarms during their shift to report accuracy and vigilance of recordings as well as place an event marker in response to the poor patient contact alarm on the NIRS monitor. We will have a laminated chart specifying treatment ‘as per medical officer’s instructions’ for the control group.

Concomitant care

There are no restrictions on concomitant medications; infant care will be as per the local NICU practice.

Outcomes

Tool	Outcomes	Time points
Cerebral NIRS monitors	Burden of hypoxia and hyperoxia expressed as percentage time spent < 65% and > 90%.	First 5 days of monitoring period
Medical records	Mortality rates	Prior to home discharge
Cerebral imaging	Evidence of brain injury on cranial ultrasounds and MRI brain (when performed for clinical reasons)	Prior to home discharge
Medical records	Presence of neonatal morbidities (chronic lung disease, necrotizing enterocolitis, retinopathy of prematurity)	Prior to home discharge
Neuro-developmental testing	• General movement assessment<br>• Cognition, motor and language domain scores on Bayley Scales of Infant	At 4 months (CGA)<br><br>At 12 months (CGA), 18-24 months (CGA), and 5 years of age.

	and Toddler development<br>• Wechsler Preschool and Primary Scale of Intelligence	5 years of age
Pulse oximeter, medical records	pulse oximetry (SpO_2), heart rate (HR) and blood pressure	First 5 days of monitoring period
Medical records	Serious and non-serious adverse reactions	First 5 days of monitoring period

7. Study Population

7.1 Recruitment Procedure

Where possible, an informed written consent will be obtained from the parent in the antenatal period. For antenatal consenting, pregnant women considered at risk of delivering before 29 weeks gestation will be approached by the research team member or a clinical team member (trained medical personnel such as neonatal fellow/registrar/consultant) who will introduce the trial to parents and provide a participant information sheet. Parents will then be able to have an informed discussion with the Research team member or clinical team member who will obtain written consent from those willing to participate in the trial. For participants where antenatal consent was obtained, randomisation will occur after birth of the child if study inclusion criteria are met.

Often premature infants deliver without much time for consent, and at times, as an emergency delivery. If there was no opportunity to obtain antenatal consent, , instead of postnatal consenting within 6 hours of birth, a waiver of consent has been approved for enrolment of the baby and consent for continued participation will be obtained in first 5 days after birth of the baby by the research team member or a clinical team member (trained medical personnel such as neonatal fellow/registrar/consultant).

7.2 Inclusion Criteria

- Preterm infants (singleton or twins births) <29 weeks gestation who are either inborn (born at the local study site hospital) or outborn (born outside the local study site hospital) and less than 6 hours of age when admitted to the study site NICU.

7.3 Exclusion Criteria

- Infants admitted to NICU beyond 6 hours of age.
- Infants with an antenatal or postnatal diagnosis of major congenital anomaly requiring major surgery or a genetic disorder associated with neurological impairment.
- Multiple births beyond twins are excluded for practical reasons (sites may not have additional NIRS monitors and the rates of multiple births beyond twins in Australia and New Zealand is very low < 2%).

7.4 Consent

Recruitment of participants will occur after obtaining an informed written consent. Where possible, an informed written consent will be obtained from the parent in the antenatal period. Where antenatal consent could not be obtained, instead of postnatal consenting within 6 hours of birth, a waiver of consent has been approved for enrolment of the baby. Families will be approached within the first five days after birth and consent will be obtained prior to the use of data.

Explicit consent will be obtained prior to any data being used and if consent has not been obtained then the data will not be used.

Participant who are randomised but cannot be approached for consent

We anticipate a small number of participants in the study for whom it is not possible to obtain consent after randomisation. This may occur due to death of the baby, or the development of a life-threatening complication. Due to the stressful nature of their baby's illness, the parents will not be in a state to provide consent and it is inappropriate to discuss information on consent with grieving parents. In such instances, no attempt will be made to contact the parents for a consent and all data collected so far be removed from the study. Should grieving parents accept continuation of intensive care treatment, then contact will be made with the parents when they are in a state of being approached for consent.

Additionally, if parent/s have COVID-19 infection at the time of delivery or in the first 5 days following the birth of the baby, they are very stressed and are in isolation for several days. These parents are not in a state to provide consent and it is inappropriate to discuss information on consent. In such instances, contact will be made with the parents when they are in a state of being approached for consent, which will usually be beyond 5 days of age.

The provision of study information may occur by email followed by a phone conversation or video conferencing with the family to facilitate timely consenting.

8. Participant Safety and Withdrawal

8.1 Risk Management and Safety

NIRS monitors have Therapeutic Goods Administration approval (for Australian sites) and Food and Drug administration approval (for US sites).

Regular skin surveillance as per local unit practice will be conducted to check the skin health from NIRS sensor placement. In our experience, when using non-adhesive NIRS sensors, skin irritation is very rare and is temporary in nature, does not require treatment and using current clinical practice (such as no tight bandages etc. use) is highly unlikely to cause any clinically relevant problems.

As a standard practice local hospital social worker routinely provide counselling to parents of premature babies. Further sessions can be offered to families who are participating in the clinical trial if needed.

8.2 Adverse Event Reporting

Serious adverse event: any adverse event that results in death, is life-threatening, requires prolongation of existing hospitalisation, result in persistent or significant disability or incapacity, or requires intervention to prevent permanent impairment or damage.

Below is a list of expected serious adverse events in premature babies born at this gestational age:

- Death before hospital discharge
- Serious early and/ late onset sepsis
- Major intra-ventricular haemorrhage
- Neonatal chronic lung disease
- Pulmonary air leaks
- Advanced necrotising enterocolitis
- Peri-ventricular leukomalacia (PVL) or diffuse white matter loss
- Advanced retinopathy of prematurity
- Sensorineural hearing loss
- Cerebral palsy
- Moderate to severe functional impairment.

Serious adverse events which may be life threatening are common in preterm infants, however, the proportion of **unexpected serious adverse events** (in the opinion of the investigator) is expected to be small.
AN UNEXPECTED SERIOUS ADVERSE EVENT(SAE) is any untoward medical occurrence that is not expected and:
- results in death, or
- is life-threatening (i.e. the subject is at risk of death at the time of the event), or
- requires prolongation of hospitalisation, or
- results in persistent or significant disability or incapacity

or
- other important medical events which, in the opinion of the investigator, are likely to become serious if untreated

NOTE: The term "life-threatening" in the definition of "serious" refers to an event in which the baby was at risk of death at the time of the event; it does not refer to an event which hypothetically might have caused death if it were more severe.

The investigator is responsible for reporting all Unexpected Serious Adverse Events occurring during the study to the CI's within 1 working day by email. The Trial Management Committee (TMC) all other Principal Investigators participating in the study will be informed. The investigator or delegate at each participating institution is responsible for reporting unexpected serious adverse events to their HREC.

An independent Data Safety and Management Committee (DSMC) will be established, the DSMC will monitor the progress of all aspects of the study and will ensure that the study meets the highest standards of ethics and patient safety. It will review interim data and other emerging evidence, including relevant RCTs and overviews of RCTs. The IDSMC will advise the TMC if in their view there is proof beyond reasonable doubt of net clinical benefit or harm, for all infants or for a subset of infants, that might reasonably be expected to influence the management of many clinicians. Data on key study outcomes will be monitored every 12 months, or more frequently if requested by the IDSMC, to ensure safety. A charter will be written and agreed upon by TMC and DSMC for stopping the study prematurely, monitoring the trial more frequently or modifying the trial design.

8.3 Handling of Withdrawals

Parents that wish to withdraw their child's participation from the trial may do so at any stage of the study, without giving a reason and without any change to any aspect of treatment or relationship with the treating medical team. Parents of any child who is withdrawn from the study after randomisation and before or after the intervention is administered will be asked to allow collection of de-identified data. In our experience, based on currently ongoing and past prospective studies, participants withdrawing after providing consent is extremely uncommon.

8.4 Replacements

Withdrawn participants will be replaced in the study. In Australia, New Zealand and other participating centres, a good neuro-developmental follow up exists as a standard practice where children born this young, are routinely followed up for 5 years and their developmental assessment is performed. To facilitate and ensure good long term follow up, existing measures which are standard practices includes providing appointment letters by post, reminding and confirming with families by phone and/or text message when follow up appointment is close to date.

9. Statistical Methods

9.1 Sample Size Estimation & Justification

A 50% reduction in mean burden of hypoxia and hyperoxia in active treatment relative to control would be considered worthwhile. This corresponds to a reduction of 0.3 in the mean of log-transformed burden. In the Safe BoosC II study (BoosC results) a 58% reduction was achieved. Assuming a standard deviation of 0.5 in log-transformed burden (Ref – BoosC protocol), this would require 45 babies per group to achieve 80% power at 5% two-sided alpha. To account for the clustering effect of twins being allocated to the same treatment, we assume that 30% of babies are twins (giving an average cluster size of 1.3, and a within-twin correlation of 0.1 in the outcome. This gives a design effect of 1.03 by which the sample size is adjusted, leading to a sample size of 47 per group, 94 babies in total. We aim to recruit 100 babies to account for some loss to follow-up or missing data.

9.2 Power Calculations

See above

9.3 Statistical Methods To Be Undertaken

Participant demographic and clinical characteristics and study outcomes will be presented using standard descriptive statistics: frequencies and percentages for categorical variables, mean, standard deviation and range or median, quartiles and range for continuous variables and the Kaplan-Meier method for time to event variables.

All efficacy analyses will be performed according to randomized treatment. The primary outcome of the log-transformed burden of hypoxia will be compared using generalized estimating equation methods with a normal distribution of the outcome and allowing for correlation between twins. The reduction in mean log-transformed burden and its 95% confidence limits will be back-transformed to calculate percentage reduction in mean burden and its confidence interval.

Other study outcomes will be compared using similar methods, using GEE methods with normal or binary outcome distribution and correlation between outcomes in twins. Adjusted models will explore predictors out outcomes. A detailed statistical analysis plan will be prepared prior to un-blinding of study results.

10. Storage of Blood and Tissue Samples

10.1 Details of where samples will be stored, and the type of consent for future use of samples

Not applicable as blood and tissue samples are not collected for this study.

11. Data Security & Handling

11.1 Details of where records will be kept & How long will they be stored

Local data for local participants will be stored with local PI's

Central team (CI's, trial coordinator) will have access to de-identified data for all participants.

Data will be stored for a minimum of 15 years or until the youngest participant turns 25 (whichever is the longest).

11.2 Confidentiality and Security

The study will be conducted in accordance with applicable Privacy Acts and Regulations. All data generated in this study will remain confidential. All consent forms and identifiable information will be stored in a separate, locked filing cabinet or as per local PI's arrangement. Data collected at each study site will be coded and stored in a locked filing cabinet and electronically (password protected) as per local site security arrangements. All measurable steps will be taken to ensure that health information collected is protected at all times. Any identification codes will be stored in a different place from the data records to which they are linked. Data management will comply with relevant privacy protocols, such as The Australian Standard on personal privacy protection.

REDCap will maintain a list of participants enrolled. Each site PI will have access only to their site records. The three chief investigators and one clinical trial coordinator will have access to all sites de-identified data to oversee enrollment. Participants will be given unique codes to protect their identity on all documentation utilised for recording and analysis purposes. Separate data records will be maintained for identifiable and de-identified data. Site PI will be responsible for collating and maintaining local site data. For each site, the local PI or research team will download continuous data such as NIRS, physiological (peripheral oxygen saturation, heart rate) data and store it electronically using password protection on work computers. Each participant will be coded based on the centre. This data will be downloaded using SenSmart™ data management software into a CSV format which will then be exported to a statistical software for data analysis. Cranial ultrasounds and MRI brain (when performed) will be de-identified (where possible) and transferred electronically for central reporting by the Neuro-radiologists. This data will remain

with the Neuro-Radiologist securely at The Children's Hospital at Westmead, Radiology Department. All data files (de-identified) will be emailed to the CI's using work emails only. Motor performance videos will be transferred for centralised scoring using Accellion Secure File Transfer endorsed by NSW Health.

Following completion of this trial, a larger study is being planned. TMC and DMSC will consider if participant information can be used for the larger trial if there is no major change to study methodology.

Secure data disposal: After completion of the storage period, paper-based records will be disposed of securely in the respective institution's confidential disposal bins (red bins in NSW). All electronically stored data will be permanently deleted.

11.3 Ancillary Data

Short videos (2-3 minutes long) are routinely collected to perform motor performance testing as part of routine developmental follow up. This will be kept as per local PI's arrangement in workplace computer that is password protected. Local PI will maintain record of this video (coded) and transfer using a secure electronic file transfer system approved by NSW Health Department or local health authority (such as Accellion Secure File Transfer). After the central reporting team has performed motor performance reporting, the de-identified video will be deleted by the central reporting team. Local PI will continue to retain a copy for medical records.

12. Appendix

List of Attachments included:

Document Name	Version Number	Date (e.g., 18 January 2012)
Picture of NIRS sensor on manikin	1	25/10/2020

13. References

1. Born too soon: the global action report on preterm birth. Geneva: World Health Organization; 2012.

2. Blencowe H, Cousens S, Oestergaard MZ, Chou D, Moller A-B, Narwal R, et al. National, regional, and worldwide estimates of preterm birth rates in the year 2010 with time trends since 1990 for selected countries: a systematic analysis and implications. The lancet. 2012;379(9832):2162-72.

3. Liu L, Johnson HL, Cousens S, Perin J, Scott S, Lawn JE, et al. Global, regional, and national causes of child mortality: an updated systematic analysis for 2010 with time trends since 2000. The Lancet. 2012;379(9832):2151-61.

4. Chow SSW, Creighton P, Chambers GM, Lui K. Report of the Australian and New Zealand Neonatal Network 2017. Sydney:: ANZNN; 2019.

5. Spittle AJ, Cameron K, Doyle LW, Cheong JL. Motor Impairment Trends in Extremely Preterm Children: 1991–2005. Pediatrics. 2018;141(4):e20173410.

6. The Economic Impact of Cerebral Palsy in Australia in 2007. Access Economics Pty Ltd; 2008.

7. Askie LM, Henderson-Smart DJ, Irwig L, Simpson JM. Oxygen-saturation targets and outcomes in extremely preterm infants. New England Journal of Medicine. 2003;349(10):959-67.

8. Darlow BA, Marschner SL, Donoghoe M, Battin MR, Broadbent RS, Elder MJ, et al. Randomized controlled trial of oxygen saturation targets in very preterm infants: two year outcomes. The Journal of pediatrics. 2014;165(1):30-5. e2.

9. Network SSGotEKSNNR. Target ranges of oxygen saturation in extremely preterm infants. New England Journal of Medicine. 2010;362(21):1959-69.

10. Schmidt B, Whyte RK, Asztalos EV, Moddemann D, Poets C, Rabi Y, et al. Effects of targeting higher vs lower arterial oxygen saturations on death or disability in extremely preterm infants: a randomized clinical trial. Jama. 2013;309(20):2111-20.

11. Stenson BJ, Tarnow-Mordi WO, Darlow BA, Simes J, Juszczak E, Askie L, et al. Oxygen saturation and outcomes in preterm infants. The New England journal of medicine. 2013;368(22):2094-104.

12. Tarnow-Mordi WO, Stenson B, Kirby A, Juszczak E, Donoghoe M, Deshpande S, et al. Outcomes of two trials of oxygen-saturation targets in preterm infants. 2016.

13. Vaucher YE, Peralta-Carcelen M, Finer NN, Carlo WA, Gantz MG, Walsh MC, et al. Neurodevelopmental outcomes in the early CPAP and pulse oximetry trial. New England Journal of Medicine. 2012;367(26):2495-504.

14. Garvey AA, Dempsey EM. Applications of near infrared spectroscopy in the neonate. Current opinion in pediatrics. 2018;30(2):209-15.

15. Hyttel-Sorensen S, Kleiser S, Wolf M, Greisen G. Calibration of a prototype NIRS oximeter against two commercial devices on a blood-lipid phantom. Biomedical optics express. 2013;4(9):1662-72.

16. Hyttel-Sorensen S, Pellicer A, Alderliesten T, Austin T, van Bel F, Benders M, et al. Cerebral near infrared spectroscopy oximetry in extremely preterm infants: phase II randomised clinical trial. BMJ : British Medical Journal. 2015;350:g7635.

17. Alderliesten T, Dix L, Baerts W, Caicedo A, van Huffel S, Naulaers G, et al. Reference values of regional cerebral oxygen saturation during the first 3 days of life in preterm neonates. Pediatric Research. 2015;79:55.

18. Hyttel-Sorensen S, Greisen G, Als-Nielsen B, Gluud C. Cerebral near-infrared spectroscopy monitoring for prevention of brain injury in very preterm infants. Cochrane Database of Systematic Reviews. 2017(9).

19. Bernardo J, Nowacki A, Martin R, Fanaroff JM, Hibbs A. Multiples and parents of multiples prefer same arm randomization of siblings in neonatal trials. J Perinatol. 2015;35(3):208.

20. Cantagrel S, Cloarec S, Sue A, Chamboux C, Tessier V, Saliba E, et al. Consequences of pulmonary inflations (sighs) on cerebral haemodynamics in neonates ventilated by high-frequency oscillation. Acta Pædiatrica. 1999;88(9):1004-8.

21. de Waal KA, Evans N, Osborn DA, Kluckow M. Cardiorespiratory effects of changes in end expiratory pressure in ventilated newborns. Archives of Disease in Childhood-Fetal and Neonatal Edition. 2007;92(6):F444-F8.

22. Schulze A, Whyte RK, Way RC, Sinclair JC. Effect of the arterial oxygenation level on cardiac output, oxygen extraction, and oxygen consumption in low birth weight infants receiving mechanical ventilation. The Journal of pediatrics. 1995;126(5):777-84.

23. Trang T, Tibballs J, Mercier J-C, Beaufils F. Optimization of oxygen transport in mechanically ventilated newborns using oximetry and pulsed Doppler-derived cardiac output. Crit Care Med. 1988;16(11):1094-7.

24. Dietz V, Wolf M, Keel M, Siebenthal K, Baenziger O, Bucher H-U. CO2 reactivity of the cerebral hemoglobin concentration in healthy term newborns measured by near infrared spectrophotometry. Neonatology. 1999;75(2):85-90.
25. Dix LML, Weeke LC, de Vries LS, Groenendaal F, Baerts W, van Bel F, et al. Carbon dioxide fluctuations are associated with changes in cerebral oxygenation and electrical activity in infants born preterm. The Journal of pediatrics. 2017;187:66-72. e1.
26. Evans N, Kluckow M. Early determinants of right and left ventricular output in ventilated preterm infants. Archives of Disease in Childhood - Fetal and Neonatal Edition. 1996;74(2):F88-F94.
27. Jim W-T, Chiu N-C, Chen M-R, Hung H-Y, Kao H-A, Hsu C-H, et al. Cerebral hemodynamic change and intraventricular hemorrhage in very low birth weight infants with patent ductus arteriosus. Ultrasound in medicine & biology. 2005;31(2):197-202.
28. Kissack CM, Garr R, Wardle SP, Weindling AM. Cerebral fractional oxygen extraction in very low birth weight infants is high when there is low left ventricular output and hypocarbia but is unaffected by hypotension. Pediatric research. 2004;55(3):400.
29. Lemmers PM, Toet MC, van Bel F. Impact of patent ductus arteriosus and subsequent therapy with indomethacin on cerebral oxygenation in preterm infants. Pediatrics. 2008;121(1):142-7.
30. Moran M, Miletin J, Pichova K, Dempsey E. Cerebral tissue oxygenation index and superior vena cava blood flow in the very low birth weight infant. Acta Pædiatrica. 2009;98(1):43-6.
31. Osborn D, Evans N, Kluckow M. Randomized trial of dobutamine versus dopamine in preterm infants with low systemic blood flow. The Journal of pediatrics. 2002;140(2):183-91.
32. Osborn DA, Evans N, Kluckow M, Bowen JR, Rieger I. Low Superior Vena Cava Flow and Effect of Inotropes on Neurodevelopment to 3 Years in Preterm Infants. Pediatrics. 2007;120(2):372-80.
33. Pellicer A, del Carmen Bravo M, Madero R, Salas S, Quero J, Cabañas F. Early Systemic Hypotension and Vasopressor Support in Low Birth Weight Infants: Impact on Neurodevelopment. Pediatrics. 2009;123(5):1369-76.
34. Pellicer A, Valverde E, Elorza MD, Madero R, Gayá F, Quero J, et al. Cardiovascular Support for Low Birth Weight Infants and Cerebral Hemodynamics: A Randomized, Blinded, Clinical Trial. Pediatrics. 2005;115(6):1501-12.
35. Pryds O, Greisen G, Skov L, Friis-Hansen B. Carbon dioxide-related changes in cerebral blood volume and cerebral blood flow in mechanically ventilated preterm neonates: comparison of near infrared spectrophotometry and 133 Xenon clearance. Pediatric research. 1990;27(5):445.
36. Skinner JR, Boys RJ, Hunter S, Hey EN. Pulmonary and systemic arterial pressure in hyaline membrane disease. Arch Dis Child. 1992;67(4 Spec No):366-73.
37. Takami T, Sunohara D, Kondo A, Mizukaki N, Suganami Y, Takei Y, et al. Changes in cerebral perfusion in extremely LBW infants during the first 72 h after birth. Pediatric research. 2010;68(5):435.
38. Vanderhaegen J, Naulaers G, Vanhole C, De Smet D, Van Huffel S, Vanhaesebrouck S, et al. The effect of changes in tPCO2 on the fractional tissue oxygen extraction–as measured by near-infrared spectroscopy–in neonates during the first days of life. european journal of paediatric neurology. 2009;13(2):128-34.
39. Andersen CC, Karayil SM, Hodyl NA, Stark MJ. Early red cell transfusion favourably alters cerebral oxygen extraction in very preterm newborns. Archives of Disease in Childhood Fetal & Neonatal Edition. 2015;100(5):F433-F5.
40. Baenziger O, Stolkin F, Keel M, von Siebenthal K, Fauchere J-C, Kundu SD, et al. The influence of the timing of cord clamping on postnatal cerebral oxygenation in preterm neonates: a randomized, controlled trial. Pediatrics. 2007;119(3):455-9.
41. Dani C, Pezzati M, Martelli E, Prussi C, Bertini G, Rubaltelli FF. Effect of blood transfusions on cerebral haemodynamics in preterm infants. Acta Paediatr. 2002;91(9):938-41.
42. Dani C, Pratesi S, Fontanelli G, Barp J, Bertini G. Blood transfusions increase cerebral, splanchnic, and renal oxygenation in anemic preterm infants. Transfusion. 2010;50(6):1220-6.
43. Wardle SP, Yoxall CW, Weindling AM. Determinants of cerebral fractional oxygen extraction using near infrared spectroscopy in preterm neonates. Journal of Cerebral Blood Flow & Metabolism. 2000;20(2):272-9.

44. Chow LC, Wright KW, Sola A. Can changes in clinical practice decrease the incidence of severe retinopathy of prematurity in very low birth weight infants? Pediatrics. 2003;111(2):339-45.
45. Collins MP, Lorenz JM, Jetton JR, Paneth N. Hypocapnia and other ventilation-related risk factors for cerebral palsy in low birth weight infants. Pediatric research. 2001;50(6):712.
46. Tin W, Milligan D, Pennefather P, Hey E. Pulse oximetry, severe retinopathy, and outcome at one year in babies of less than 28 weeks gestation. Archives of Disease in Childhood-Fetal and Neonatal Edition. 2001;84(2):F106-F10.
47. Vesoulis ZA, Lust CE, Liao SM, Trivedi SB, Mathur AM. Early hyperoxia burden detected by cerebral near-infrared spectroscopy is superior to pulse oximetry for prediction of severe retinopathy of prematurity. J Perinatol. 2016;36(11):966-71.
48. Pryds O, Christensen N, Friis-Hansen B. Increased cerebral blood flow and plasma epinephrine in hypoglycemic, preterm neonates. Pediatrics. 1990;85(2):172-6.
49. Skov L, Pryds O. Capillary recruitment for preservation of cerebral glucose influx in hypoglycemic, preterm newborns: evidence for a glucose sensor? Pediatrics. 1992;90(2):193-5.

STATISTICAL ANALYSIS PLAN

Near **I**nfra-**R**ed spectroscopy **T**argeted **U**se to **R**educe adverse outcomes in **E**xtremely preterm infants -NIRTURE trial

Protocol version 1.12 dated 14.12.2022.

The Australian New Zealand Clinical Trials Registry, registration number: ACTRN12621000778886

STUDY SYNOPSIS

The NIRTURE trial is a phase II single-blinded RCT with two parallel groups with 1:1 allocation stratified for site and gestational age (<26 weeks and $\geq 26^{+0}$–28^{+6} weeks' gestation) addressing the following question: In premature babies (< 29 weeks gestation at birth), can dedicated treatment with cerebral oxygenation monitoring compared to usual treatment with blinded cerebral oxygenation monitoring, reduce the burden of cerebral hypoxia and hyperoxia in the first 5 days? The trial will use a neonatal population specific sensor and near infrared spectroscopy device from a single manufacturer.

Study design

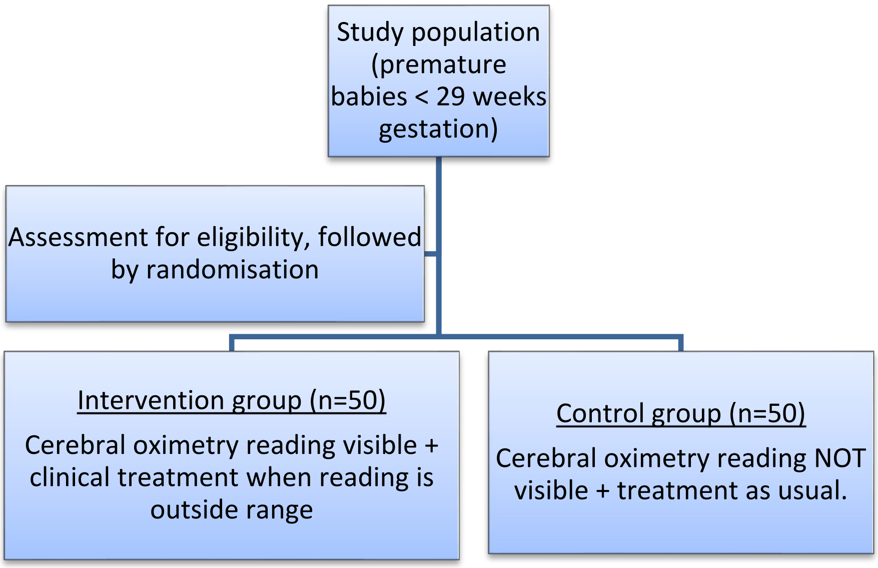

STUDY OBJECTIVES

Primary Objectives

In premature babies (< 29 weeks gestation at birth), can a dedicated treatment with cerebral oxygenation monitoring compared to usual treatment, reduce the burden of cerebral hypoxia and hyperoxia in the first 5 days?

We hypothesise that the burden of hypoxia and hyperoxia can be reduced (by half) by a combination of specific treatment guideline and keeping cerebral oxygenation in the specific range of 65-90%.

Secondary Objectives

To explore the relationship between cerebral hypoxia and hyperoxia in the first 5 days and the following:

- Mortality before hospital discharge.
- Brain injury before hospital discharge.
- Neonatal morbidities (chronic lung disease, necrotizing enterocolitis, retinopathy of prematurity) before hospital discharge.
- Neuro-developmental outcomes until 5 years of age.
- Physiological functions (heart rate, pulse oximetry, blood pressure) in the first 5 days.
- Physiological functions (observation of pulse oximetry) at 36-37 weeks PMA.
- Sleep architecture (based on sleep study) at 36-37 weeks PMA.

PATIENT POPULATION

Inclusion criteria

- Preterm infants (singleton or twin births) <29 weeks gestation who are either inborn (born at the local study site hospital) or outborn (born outside the local study site hospital) and less than 6 hours of age when admitted to the study site NICU.

Exclusion criteria

- Infants admitted to NICU beyond 6 hours of age.
- Infants with an antenatal or postnatal diagnosis of major congenital anomaly requiring major surgery or a genetic disorder associated with neurological impairment.
- Multiple births beyond twins are excluded for practical reasons (sites may not have additional NIRS monitors and the rates of multiple births beyond twins in Australia and New Zealand is very low < 2%).

OUTCOMES

Primary outcome

- The burden of cerebral hypoxia and hyperoxia during the first 5 days expressed as percent hours.

Secondary outcomes

- Newborn mortality rates prior to home discharge
- Brain injury based on cerebral imaging prior to home discharge
- Neonatal morbidities (chronic lung disease, necrotizing enterocolitis, retinopathy of prematurity) prior to home discharge

- Physiological functions (observation of pulse oximetry) at 36-37 weeks PMA.
- Sleep architecture (based on sleep study) at 36-37 weeks PMA.
- Motor performance assessment (General movements assessment) during NICU stay and at 4 months corrected age follow up.
- Neuro-developmental outcomes until 5 years of age.
- Measurement of physiological functions (heart rate, pulse oximetry, blood pressure) in the first 5 days.

Safety outcomes

- Skin injury (pressure or thermal) from NIRS sensor in the first 5 days after birth and adverse event reporting.

INTERVENTION

After enrolment, infants will be randomised to either control group or intervention group. Monitoring of cerebral oxygenation will be commenced by using SenSmart™ Model X-100 Universal Oximetry System (from NONIN Medical Inc, MN, US) and placing a neonatal NIRS sensor on the fronto-parietal region of the infant's forehead and monitoring will be continued for 5 days (120 hours of monitoring). For the Intervention group, cerebral oxygenation reading will be visible, and the infant will be treated according to a dedicated clinical treatment algorithm when the cerebral oxygenation is outside the range of 65%–90% (see protocol).

Intervention thresholds

A pragmatic consensus-based approach was used for selecting 65%–90% as the reference range for cerebral oxygenation. The lower threshold of 65% was chosen based on the lower threshold (55% using an adult sensor) used by the SafeBoosC-II study and higher absolute oxygenation values with neonatal sensors. A upper threshold value of 90% instead of 85% (as selected by the SafeBoosC-II study) was chosen from our local, unpublished data by monitoring preterm infants born <28 weeks gestation in the first three days of life with NONIN neonatal sensors (cerebral oxygenation of 87% was the 75th centile). The sensor site will be inspected every 4 hours to ensure correct sensor placement and skin integrity. Bedside clinicians will receive training on application of NIRS monitoring, troubleshooting advice for sensor disconnection and for technical issues.

A clinical treatment algorithm for cerebral hypoxia is triggered when cerebral oxygenation, CrSO2 is <65% (see protocol). When Cerebral oxygenation (CrSO2) is <65% potential causes include inaccurate sensor placement, low arterial oxygen saturation, reduced cerebral blood flow or reduced haemoglobin concentration in circulating blood. A clinical treatment algorithm for cerebral hyperoxia is triggered when cerebral oxygenation, CrSO2 is >90% (see protocol). When cerebral oxygenation (CrSO2) is >90% potential causes include inaccurate sensor placement, high arterial oxygen saturation, increased cerebral blood flow, or hypoglycaemia.

For the control group, cerebral oxygenation reading will not be visible to the bedside staff and the infant will be treated according to standard clinical practice. We will perform blinded NIRS monitoring by covering the monitor screen with an opaque cover and the information will not be available to clinicians to act upon.

RANDOMISATION AND BLINDING

Central randomisation will be performed by the site investigator or the medical team member using REDCap electronic data capture tools hosted at University of Sydney. Infants assessed as eligible will be randomised to control group or intervention group by using variable block randomisation stratified for gestation age (<26 weeks' and ≥26 weeks') and study site. Randomisation codes for the study will be generated by a biostatistician independent to the study. Permuted blocks will maintain group balance, infants will be allocated to two groups in a 1:1 ratio. Twins will be randomised to the same group, based on negligible intra-class coefficient burden of hypoxia within pairs of twins in SafeBoosC-II study and survey of parents of multiple births.

To ensure concealment, the block sizes will not be disclosed. Additionally, allocation concealment will be ensured, as REDCap will not reveal the randomisation until the infant has been recruited into the trial. REDCap will maintain a list of participants enrolled. Each site investigator/s will access only to their site records. The chief investigators will have access to all sites to oversee enrolment. Participants will be given unique codes to protect their identity on all documentation utilised for recording and analysis purposes. Separate data records will be maintained for re-identifiable and de-identified data.

Blinding (masking)

During the intervention period, the assignment of infants to control arm or intervention arm cannot be blinded to the clinical staff. For infants in the control arm, blinding will be performed by covering the NIRS monitor screen with an opaque cover. Also, alarms for the range of cerebral oxygenation (65%–90%) will be turned off and alarms for displacement/dislodgement of the sensors will remain active. The statistician and neurodevelopment outcome assessors will be blinded to the intervention.

SAMPLE SIZE

Based on the results of the Safe-BoosC-II study, a 50% reduction in mean burden of hypoxia and hyperoxia in active treatment relative to control would be considered worthwhile. This corresponds to a reduction of 0.3 in the mean of log-transformed burden of cerebral hypoxia and/or hyperoxia. In the Safe BoosC-II study, a 58% reduction was achieved. Assuming a standard deviation of 0.5 in log-transformed burden, this would require 45 babies per group to achieve 80% power at 5% two-sided alpha. To account for the clustering effect of twins being allocated to the same treatment, we assume that 30% of babies are twins-based on regional incidence of twin births (giving an average cluster size of 1.3, and a within-twin correlation of 0.1 in the outcome. This gives a design effect of 1.03 by which the sample size is adjusted, leading to a sample size of 47 per group, 94 babies in total. We aim to recruit 100 babies to account for some loss to follow-up or missing data. For consented participants, if the NIRS monitoring was discontinued earlier than 5 days after birth, data collected until discontinuation of monitoring will be used for analysis of the primary outcome.

STATISTICAL ANALYSIS

Analysis will be conducted per-protocol using RStudio.Version(4) at a significance level of 0.05 with a two-sided alternative hypothesis. The reporting will adhere to the CONSORT guidelines.

Data cleaning: In absence of international guidelines on handling NIRS artifacts, the artifacts will be included in the data analysis. Extreme values for cerebral oxygenation (0 and 100) will be removed.

Interim analysis

There will not be an interim analysis. The Data and Safety Monitoring Board's first meeting will be 6 months from initial recruitment and will continue every 6 monthly until end of recruitment. A review of the study by the data and safety monitoring board will be performed.

Analysis of the outcomes

Participant demographic and clinical characteristics and study outcomes will be presented using standard descriptive statistics: frequencies and percentages for categorical variables, mean, standard deviation and range or median, quartiles and range for continuous variables. All efficacy analyses will be performed according to randomized treatment. We will use a suitable transformation to ensure normality for the primary outcome, the burden of cerebral hypoxia and hyperoxia and use generalized estimating equation (GEE) or a similar method for comparison between groups. Sub-group analysis for stratification variables will be performed. For reporting the burden of cerebral hypoxia and hyperoxia, we will include deviations in cerebral oxygenation more than a minute. This pragmatic approach will ensure we achieve a balance between responding to an alarm from poor sensor contact with the participant and identifying true real-time changes in cerebral oxygenation. To allow comparison of the primary outcomes with other studies, we will also report the burden of cerebral hypoxia and hyperoxia more than 10 minutes. Other study outcomes will be compared using similar methods, using GEE methods with normal or binary outcome distribution and correlation between outcomes in twins. Adjusted models will explore predictors out outcomes. No corrections for multiple hypotheses will be performed. Adjusted models will be used to explore predictors of outcomes. Variables that differed significantly between the two groups, and could plausibly contributory to the outcome, will be considered for adjustment. A fixed adjustment for gestational age category will be included as an a priori specified variable. For this trial, no imputation of missing data will be performed and sensitivity analysis will only be performed separately for the burden of cerebral hypoxia and cerebral hyperoxia. In future, sensitivity analysis may be performed for variables such as specific gestational age or treatment received.

Withdrawn participants will be replaced in the study with new randomisation (per protocol). To facilitate and ensure long term follow up, existing measures which are standard practices includes providing appointment letters by post, reminding and confirming with families by phone and/or text message when follow up appointment is close to date.

This trial will be conducted in accordance with applicable Privacy Acts and Regulations in all three countries. Data will be stored for a minimum of 15 years or until the youngest participant turns 25 (whichever is the longest).
