# Supplementary material for: Cerebral Oximetry–Guided Treatment and Cerebral Oxygenation in Extremely Preterm Infants: A Randomized Clinical Trial
Source: JAMA Netw Open. 2026 Feb 5;9(2):e2557620. doi: 10.1001/jamanetworkopen.2025.57620 (PMC12878427; doi:10.1001/jamanetworkopen.2025.57620)
Supplement: Supplement 2. — eFigure 1. Clinical treatment algorithm for cerebral hypoxia (crSO2 <65%) eFigure 2. Clinical algorithm for cerebral hyperoxia (CrSO2 >90%) eFigure 3. Interventions for cerebral hypoxia eFigure 4. Interventions for cerebral hyperoxia eTable 1. Primary outcome using 10-min trigger interval stratified by gestational age of participants eTable 2. Treatment and complications in participants within the first 5 d after birth eFigure 5. Primary outcome by participating site and day after birth eFigure 6. Primary outcome by gestational age groups and day of age after birth eFigure 7. Primary outcome by day of age after birth [file jamanetwopen-e2557620-s002.pdf]

Supplemental Online Content

Jani PR, Goyen T-A, Balegar KK, et al. Cerebral oximetry–guided treatment and cerebral oxygenation in extremely preterm infants. *JAMA Netw Open*. 2026;9(2):e2557620. doi:10.1001/jamanetworkopen.2025.57620

eFigure 1. Clinical treatment algorithm for cerebral hypoxia (crSO2 <65%)
eFigure 2. Clinical algorithm for cerebral hyperoxia (CrSO2 >90%)
eFigure 3. Interventions for cerebral hypoxia
eFigure 4. Interventions for cerebral hyperoxia
eTable 1. Primary outcome using 10-min trigger interval stratified by gestational age of participants
eTable 2. Treatment and complications in participants within the first 5 d after birth
eFigure 5. Primary outcome by participating site and day after birth
eFigure 6. Primary outcome by gestational age groups and day of age after birth
eFigure 7. Primary outcome by day of age after birth

This supplemental material has been provided by the authors to give readers additional information about their work.

© 2026 Jani PR et al. *JAMA Network Open*.

eFigure 1: Clinical treatment algorithm for cerebral hypoxia ($CrSO_2$ < 65%)

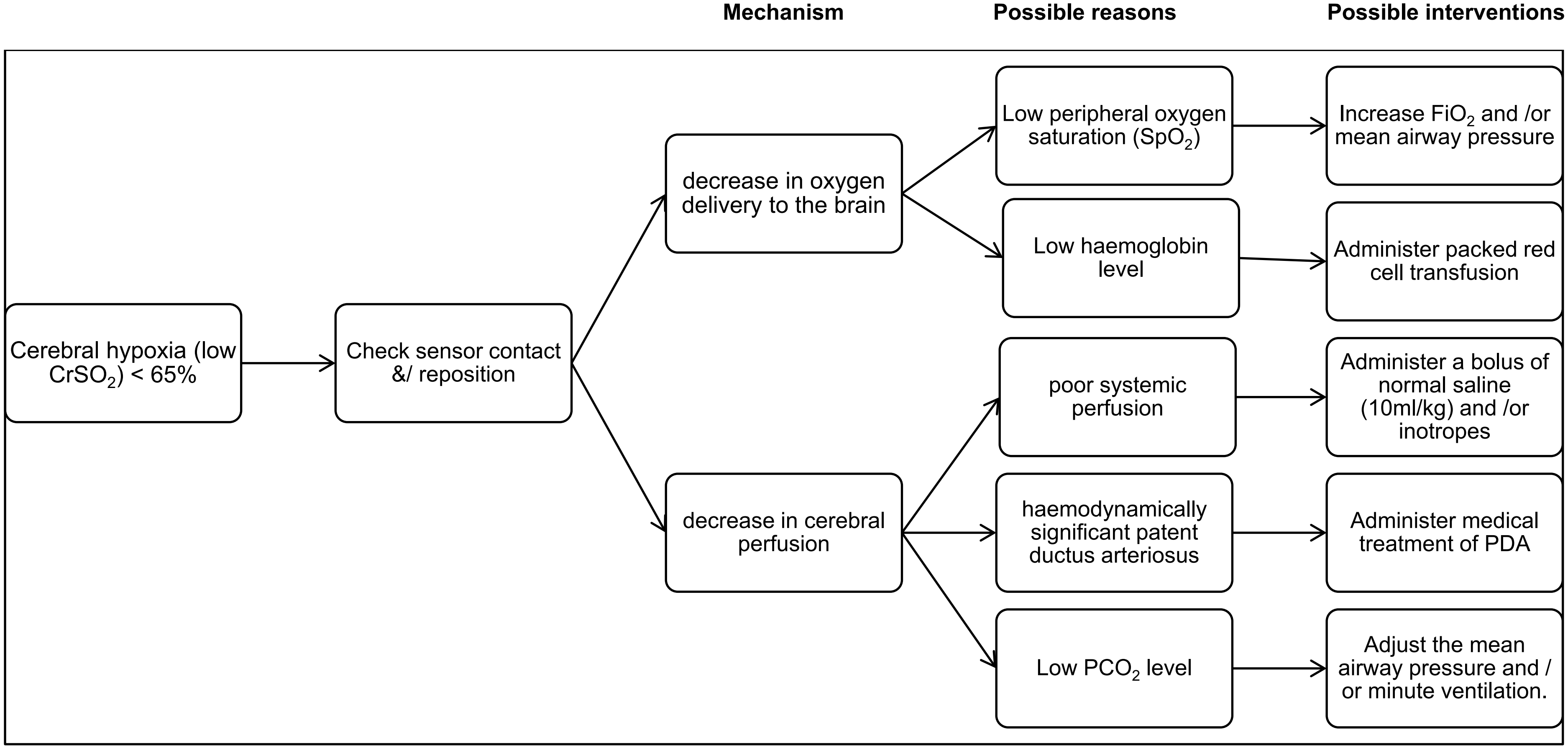

$CrSO_2$, cerebral oxygenation; FiO_2: fraction of inspired oxygen; PCO_2, partial pressure of carbon dioxide in blood; SpO_2: peripheral oxygen saturation

© 2026 Jani PR et al. *JAMA Network Open*.

eFigure 2: Clinical algorithm for cerebral hyperoxia ($CrSO_2$) is > 90%

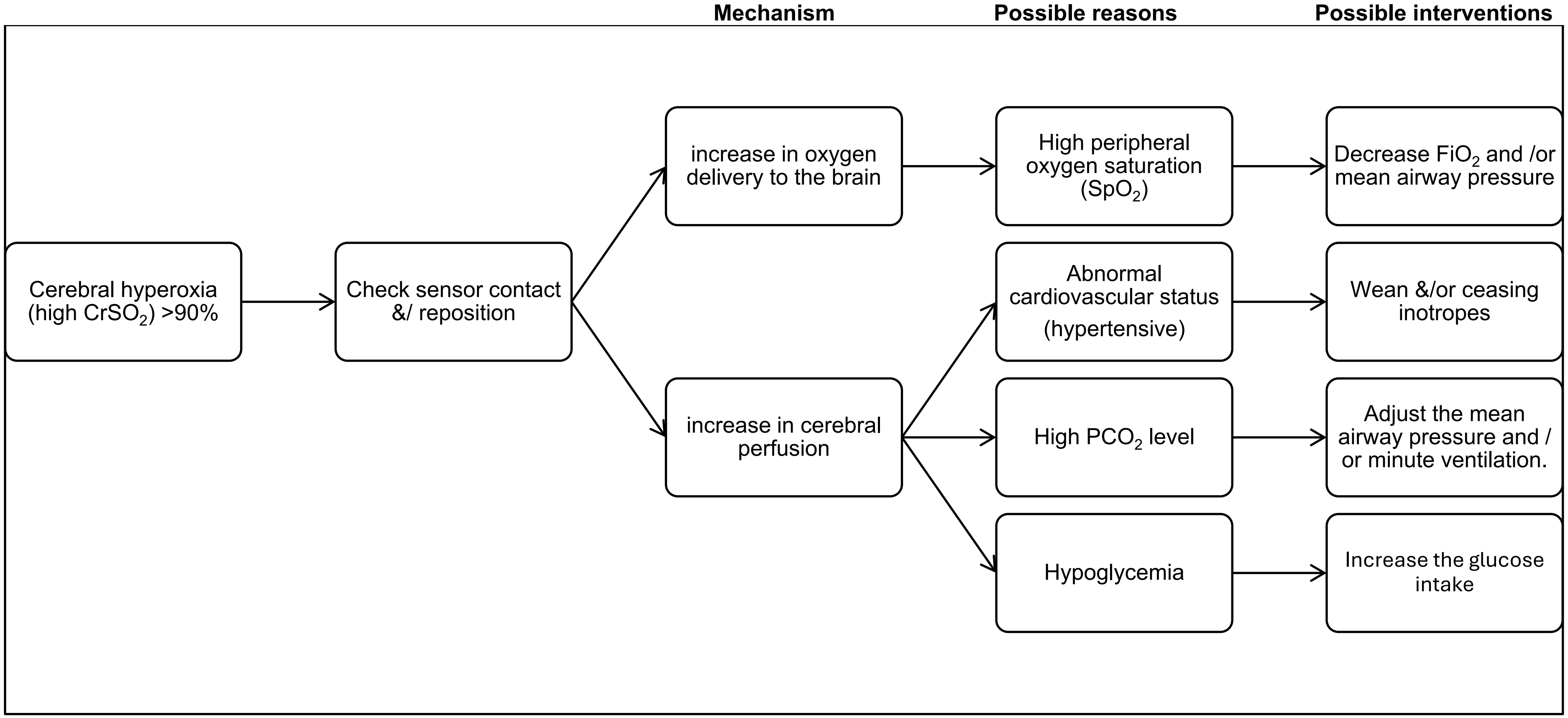

$CrSO_2$, cerebral oxygenation; FiO_2: fraction of inspired oxygen; PCO_2, partial pressure of carbon dioxide in blood; SpO_2: peripheral oxygen saturation

© 2026 Jani PR et al. *JAMA Network Open*.

eFigure 3: Interventions for cerebral hypoxia

Important causes of cerebral hypoxia (low $CrSO_2$) include (but not limited to) conditions that decrease oxygen delivery to the brain such as low SpO_2, systemic hypotension, haemodynamically significant ductus arteriosus, hypocarbia and/or anaemia).
If no change to cerebral $CrSO_2$ from sensor repositioning, and $CrSO_2$ is consistently < 65% then check for the following (perform one change at a time and reassess within 30 minutes of making the change)

- Low peripheral oxygen saturation (SpO_2): if below normal or in the low normal range then aim to bring it back to normal range by performing manoeuvres such as increasing FiO_2 and /or mean airway pressure.

- Abnormal cardiovascular status: presence of poor systemic perfusion and /or haemodynamically significant patent ductus arteriosus.
  - ✓ if clinical (capillary refill time ≥ 3 seconds or hypotension on non-invasive or invasive blood pressure measurement), or
  - ✓ biochemical (elevated serum lactate) or
  - ✓ echocardiographic signs (low cardiac output and/or low SVC flows) of poor systemic perfusion then consider administering a bolus of normal saline (10ml/kg) and /or inotropes.
  - ✓ Presence of haemodynamically significant patent ductus arteriosus (based on echocardiogram), consider medical treatment.

- Low PCO_2 level (transcutaneous or capillary/arterial blood gas): if below normal or low normal range, then aim to normalize by adjusting the mean airway pressure and / or minute ventilation.

- Low haemoglobin (Hb) level: if the most recent Hb is low consider packed red cell transfusion.

If no correctable cause for low cerebral $CrSO_2$ identified, then continue close clinical assessment.

$CrSO_2$: Cerebral oxygenation, SpO_2: peripheral oxygen saturation, SVC: superior vena cava, PCO_2: partial pressure of carbon dioxide in blood, Hb: haemoglobin

© 2026 Jani PR et al. *JAMA Network Open*.

eFigure 4: Interventions for cerebral hyperoxia

Important causes of cerebral hyperoxia (high $CrSO_2$) include (but not limited to) conditions that increase oxygen delivery to the brain such as high SpO_2, hypercarbia, systemic hypertension, and/or hypoglycemia.
If no change to cerebral $CrSO_2$ from sensor repositioning, and $CrSO_2$ is consistently >90% then check for the following (perform one change at a time and reassess within 30 minutes of making the change)

- High SpO_2: If above normal or in the high normal range, aim to bring it back to normal range by performing manoeuvres such as decreasing FiO_2 and / or mean airway pressure.
- High PCO_2 level (transcutaneous or capillary/arterial blood gas): if above normal or high normal range, then aim to normalize by adjusting the mean airway pressure and / or minute ventilation.
- Abnormal cardiovascular status: if hypertensive and receiving inotropes, considering weaning and/or ceasing inotropes.
- Hypoglycemia: if blood glucose level < 2.6 mmol/L, then increase glucose intake to maintain normal eu-glycemia.

If no cause for high cerebral $CrSO_2$ identified, then continue close clinical assessment.

$CrSO_2$: Cerebral oxygenation, SpO_2: peripheral oxygen saturation, PCO_2: partial pressure of carbon dioxide in blood

© 2026 Jani PR et al. *JAMA Network Open*.

eTable 1: Primary outcome using 10 minutes trigger interval stratified by gestational age of the participants

Outcomes	Intervention group	Standard care group	Relative change in % (95% CI)
**For Gestational ages 23^{+0} – 25^{+6}**	**n = 15**	**n = 14**	
Burden of cerebral hypoxia and hyperoxia expressed as % hours, median (interquartile range)	3.3 (2.3 – 10.9)	47.2 (34.3 – 91.7)	170.8 (107.4 to 413.6)
Burden of cerebral hypoxia expressed as % hours, median (interquartile range)	0.0 (0.0 – 0.0)	1.5 (0.0 – 11.5)	104.3 (71.3 to 187.4)
Burden of cerebral hyperoxia expressed as % hours, median (interquartile range)	3.3 (2.3 – 10.9)	47.2 (23.3 – 87.3)	154.5 (97.8 to 384.5)
**Gestational ages 26^{+0} – 28^{+6}**	**n = 35**	**n = 36**	
Burden of cerebral hypoxia and hyperoxia expressed as % hours, median (interquartile range)	4.8 (1.9 – 14.1)	19.0 (4.9 – 51.3)	59.0 (42.2 to 100.0)
Burden of cerebral hypoxia expressed as % hours, median (interquartile range)	0.0 (0.0 – 0.0)	0.0 (0.0 – 1.2)	37.2 (29.0 to 52.6)
Burden of cerebral hyperoxia expressed as % hours, median (interquartile range)	4.5 (1.9 – 14.1)	13.4 (4.2 – 44.0)	45.6 (31.2 to 88.1)
**All Gestational ages**	**n = 50**	**n = 50**	
Burden of cerebral hypoxia and hyperoxia expressed as % hours, median (interquartile range)	4.0 (2.0 – 14.4)	34.5 (6.0 – 76.5)	93.5 (68.4 to 147.4)
Burden of cerebral hypoxia expressed as % hours, median (interquartile range)	0.0 (0.0 – 0.0)	0.0 (0.0 – 2.7)	142.0 (85.8 to 414.3)
Burden of cerebral hyperoxia expressed as % hours, median (interquartile range)	3.5 (2.0 – 14.4)	23.7 (5.8 – 60.7)	80.7 (56.8 to 139.1)

© 2026 Jani PR et al. *JAMA Network Open*.

eTable 2: Treatment and complications in participants within the first 5 days after birth

	Cerebral NIRS monitoring + clinical treatment guideline (Intervention group) (n=50)	Blinded cerebral NIRS monitoring + treatment as usual (Standard care group) (n=50)
Invasive ventilation, No. (%)	36 (72)	33 (66)
Inhaled nitric oxide, No. (%)	2 (4)	2 (4)
Pulmonary air leaks, No. (%)	1 (2)	1 (2)
Postnatal systemic steroids including hydrocortisone, No. (%)	2 (4)	5 (10)
Treatment for systemic hypotension [a], No. (%)	11 (22)	15 (30)
Medical treatment for closure of patent ductus arteriosus, No. (%)	21 (42)	27 (54)
Confirmed sepsis, No. (%)	4 (8)	1 (2)
Death, No. (%)	1 (2)	1 (2)
Skin injury at sensor site, No. (%)	0	1 (2)

Abbreviation: NIRS, near infrared spectroscopy
Data is presented as number (percentage) or median (interquartile range).
[a]Normal saline bolus with or without inotropes

© 2026 Jani PR et al. *JAMA Network Open*.

eFigure 5: Primary outcome by participating site and day after birth

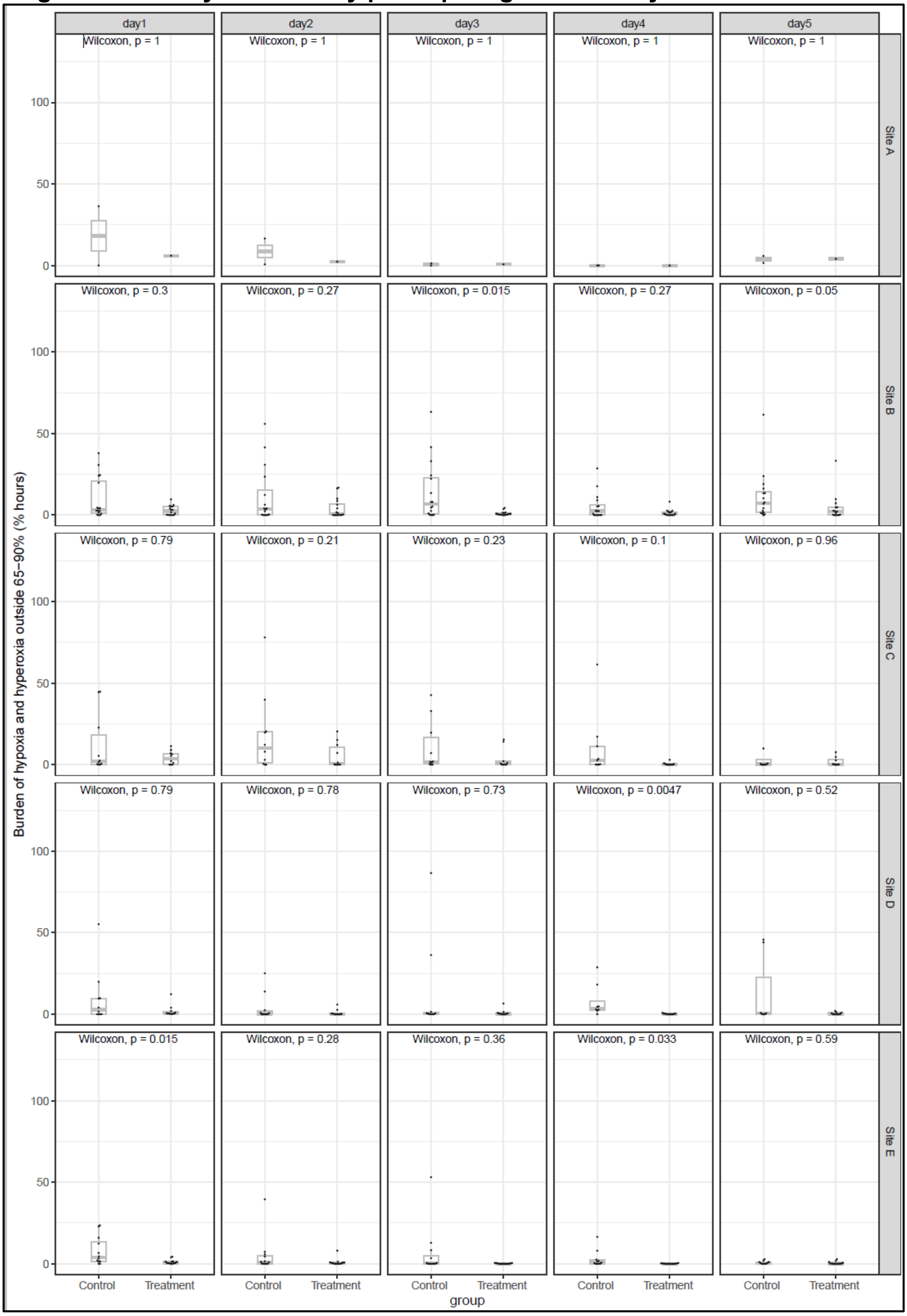

© 2026 Jani PR et al. *JAMA Network Open*.

eFigure 6: Primary outcome by gestational age groups and day of age

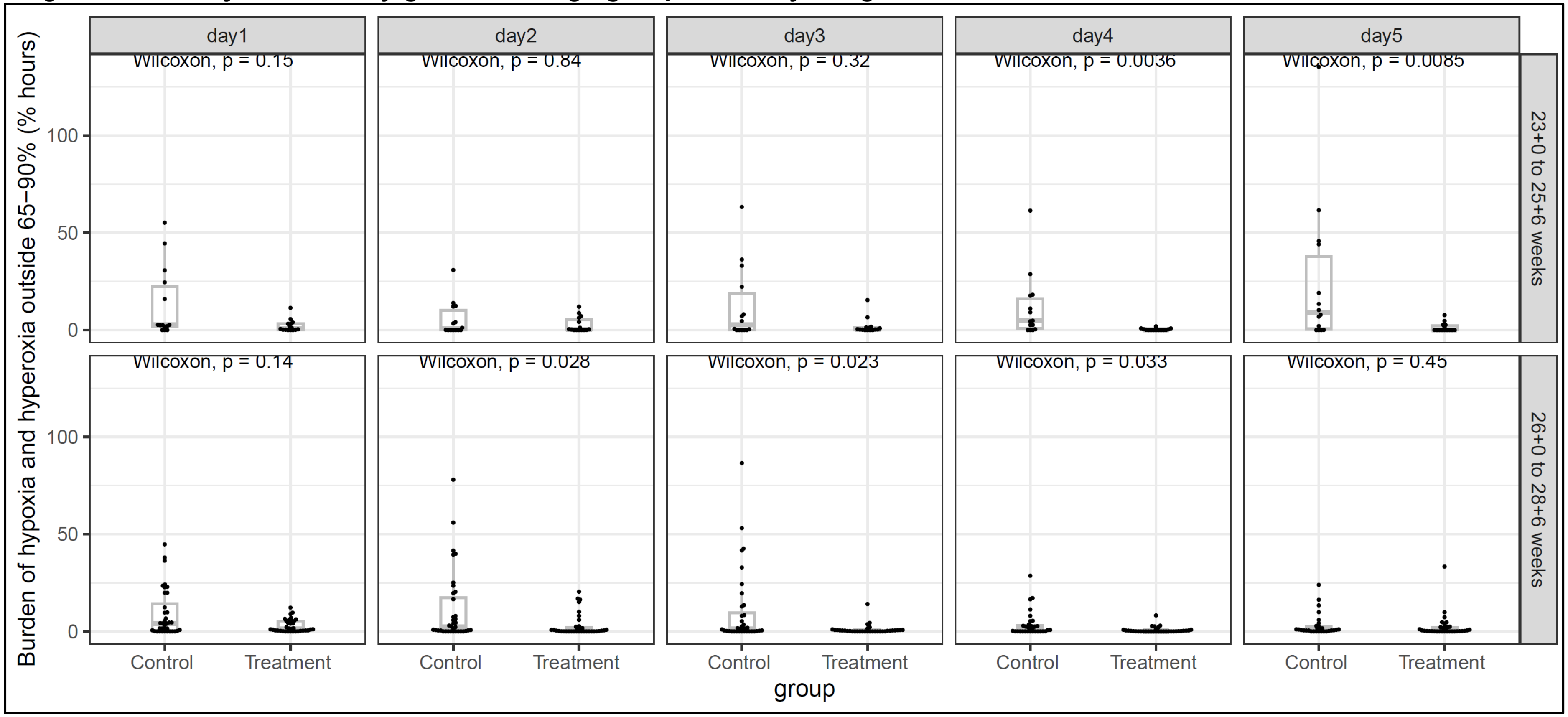

© 2026 Jani PR et al. *JAMA Network Open*.

eFigure 7: Primary outcome by day of age after birth

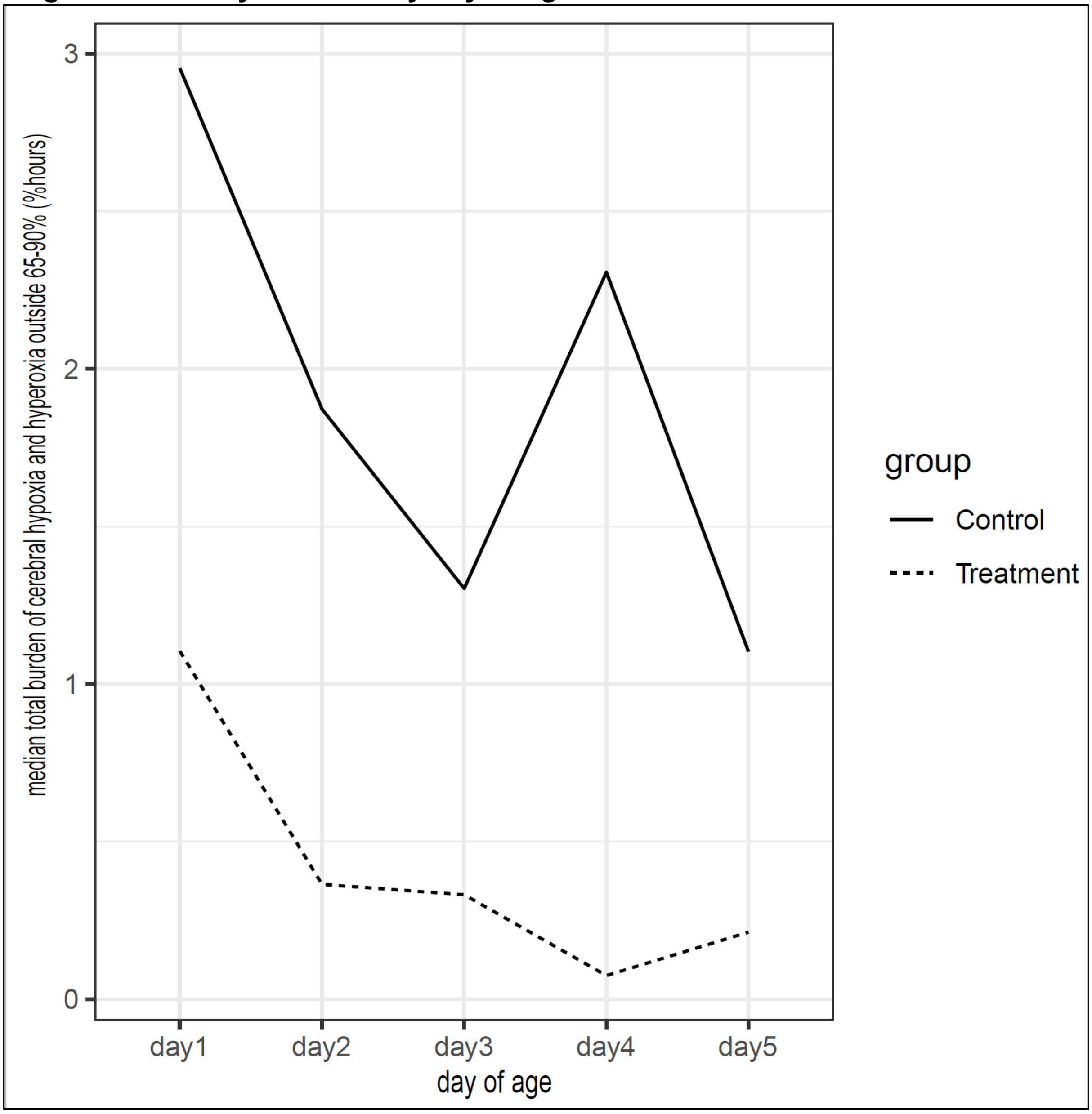

© 2026 Jani PR et al. *JAMA Network Open*.
