# Supplementary material for: Cerebral Oximetry–Guided Treatment and Cerebral Oxygenation in Extremely Preterm Infants: A Randomized Clinical Trial
Source: JAMA Netw Open. 2026 Feb 5;9(2):e2557620. doi: 10.1001/jamanetworkopen.2025.57620 (PMC12878427; doi:10.1001/jamanetworkopen.2025.57620)
Supplement: Supplement 3. — Data Sharing Statement [file jamanetwopen-e2557620-s003.pdf]

Data Sharing Statement

Jani. Cerebral Oximetry–Guided Treatment and Cerebral Oxygenation in Extremely Preterm Infants: A Randomized Clinical Trial. *JAMA Netw Open*. Published online February 5, 2026. doi:10.1001/jamanetworkopen.2025.57620

Data
Additional Information: https://www.anzctr.org.au/Trial/Registration/TrialReview.aspx?ACTRN=12621000778886
Data available: Yes
Data types: Deidentified participant data
How to access data: data: Researchers whose proposed use of the data has been approved for a specified purpose may obtain the data from Pranav.jani@health.nsw.gov.au. such request is subject to signed data access and authorship agreement with the institution's regulatory body.
When available: With publication

Supporting Documents
Document types: None

Additional Information
Who can access the data: data: Researchers whose proposed use of the data has been approved
Types of analyses: For a specific purpose
Mechanisms of data availability: Signed data access agreement
Any additional restrictions: Authorship agreement
